# Thirty years of glyphosate‐resistant crops and weeds: Current situation and future prospects

**DOI:** 10.1002/ps.70742

**Published:** 2026-03-19

**Authors:** Ricardo Alcántara‐de la Cruz, Yure Marin Guidi, Mariana Ramirez‐Castillo, Fernando Storniolo Adegas, Caio Antonio Carbonari, Edivaldo Domingues Velini, Rafael De Prado, Stephen O. Duke

**Affiliations:** ^1^ Department of Agronomy Federal University of Viçosa Viçosa Brazil; ^2^ Brazilian Agricultural Research Corporation/Embrapa Soja Londrina Brazil; ^3^ Department of Plant Protection, School of Agriculture São Paulo State University (UNESP) Botucatu Brazil; ^4^ Department of Biochemistry and Molecular Biology University of Cordoba, UCO‐CeiA3 Cordoba Spain; ^5^ National Center for Natural Products Research, School of Pharmacy, University of Mississippi Oxford MS USA

**Keywords:** 5‐enolpyruvylshikimate‐3‐phosphate synthase, glyphosate‐resistant crops, GMO, mechanism of action, multiple resistance, non‐target‐site resistance, target‐site resistance

## Abstract

Glyphosate, a broad‐spectrum herbicide targeting 5‐enolpyruvylshikimate‐3‐phosphate synthase, was first commercialized in 1974. Its use increased significantly following the introduction of glyphosate‐resistant (GR) crops in 1996 and the expiration of its patent in 2000, making it the most used herbicide worldwide. Intensive reliance on glyphosate has selected for GR weed populations, with the first documented case reported also in 1996. Glyphosate resistance now has evolved in 62 weed species across 31 countries, often involving multiple other herbicide modes of action and thereby complicating chemical control. The highest incidence has occurred in regions with extensive GR crop cultivation, particularly the United States, Brazil, and Argentina, where glyphosate has been used for years as the sole or major herbicide. Apparent disparities among countries largely reflect differences in the timing and reporting of first unique resistance cases, rather than actual differences in the overall prevalence of GR weeds. GR weeds possess the greatest diversity of resistance mechanisms described for any herbicide, encompassing both target‐site and non‐target‐site resistance mechanisms, frequently in combination. In many cases, resistance traits that provide low resistance levels have combined over time to provide more robust resistance (creeping resistance). Evolution and spread of glyphosate resistance are influenced by frequency of use, herbicide dose, phenological stage, pollination biology, gene flow, and dissemination of GR seeds or propagules via contaminated seed/grain lots and machinery. Glyphosate resistance and controversies regarding environmental and health issues have so far not resulted in marked reductions in glyphosate use nor in the introduction of a herbicide or other technology that is as effective and economical as glyphosate in managing weeds. This review synthesizes current knowledge of glyphosate resistance and its mechanisms, highlighting existing gaps, and discusses potential scenarios for the future of glyphosate and GR crops. © 2026 The Author(s). *Pest Management Science* published by John Wiley & Sons Ltd on behalf of Society of Chemical Industry.

## Graphical abstract

Since 1996, when the first glyphosate‐resistant crop was commercialized and the first resistant weed was reported, resistance has expanded globally. This review analyzes emergence patterns across weed species, crops, regions, resistance mechanisms, and herbicides. Despite widespread resistance via diverse target‐site and non‐target‐site mechanisms, glyphosate remains the most extensively used herbicide worldwide.

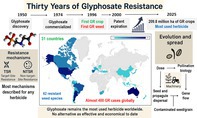

## 1 INTRODUCTION

Glyphosate (*N*‐phosphonomethyl glycine) is the only herbicide in the substituted glycine chemical group.^1^ It is used exclusively as a post‐emergence herbicide for the non‐selective control of weeds.^2^, ^3^ Due to its tight binding to soil components, it cannot be used as a soil‐applied or soil‐incorporated herbicide. Discovered as a chemical in 1950, it was initially recognized for its properties as a chelating agent, pH reducer, and detergent. The molecule was synthesized by the Swiss company Cilag/Ciba during research on chelating agents for dyes. In the 1960s, scientists at Stauffer Chemicals also identified its chelating capacity; however, its herbicidal activity was discovered in the early 1970s by Monsanto researchers.^4^ Since its commercial release in 1974, glyphosate has become a cornerstone of chemical weed control because of its broad‐spectrum activity against annual and perennial weeds, high efficacy, ease of use, low cost, and rapid environmental degradation.^5^, ^6^

Glyphosate is the only herbicide that inhibits 5‐enolpyruvylshikimate‐3‐phosphate synthase (EPSPS), a chloroplast‐localized enzyme of the shikimic pathway that is highly conserved across plant species,^3^ yet structurally distinct from EPSPS enzymes found in most bacteria. Animals lack EPSPS because they do not possess the shikimate pathway. EPSPS catalyzes the reaction between shikimate‐3‐phosphate (S3P) and phosphoenolpyruvate (PEP) in the shikimic pathway, producing EPSP and inorganic phosphate. The shikimic pathway accounts for 20% or more of carbon flow in plants and is essential for the production of aromatic amino acids (phenylalanine, tyrosine, and tryptophan).^7^ Structurally resembling PEP, glyphosate acts as a competitive inhibitor of EPSPS, thereby blocking the production of these amino acids and their essential derivatives (e.g., plastoquinone and indole acetic acid), as well as secondary metabolites from the pathway such as lignin, flavonoids, isoflavones, and anthocyanins. This metabolic disruption ultimately leads to cell death.^7^, ^8^ Although the high accumulation of shikimic and quinic acids following EPSPS inhibition has been reported to contribute to glyphosate phytotoxicity,^3^, ^9^, ^10^ their relative contribution to the overall herbicidal effect of glyphosate remains unclear.

**Figure 1:**
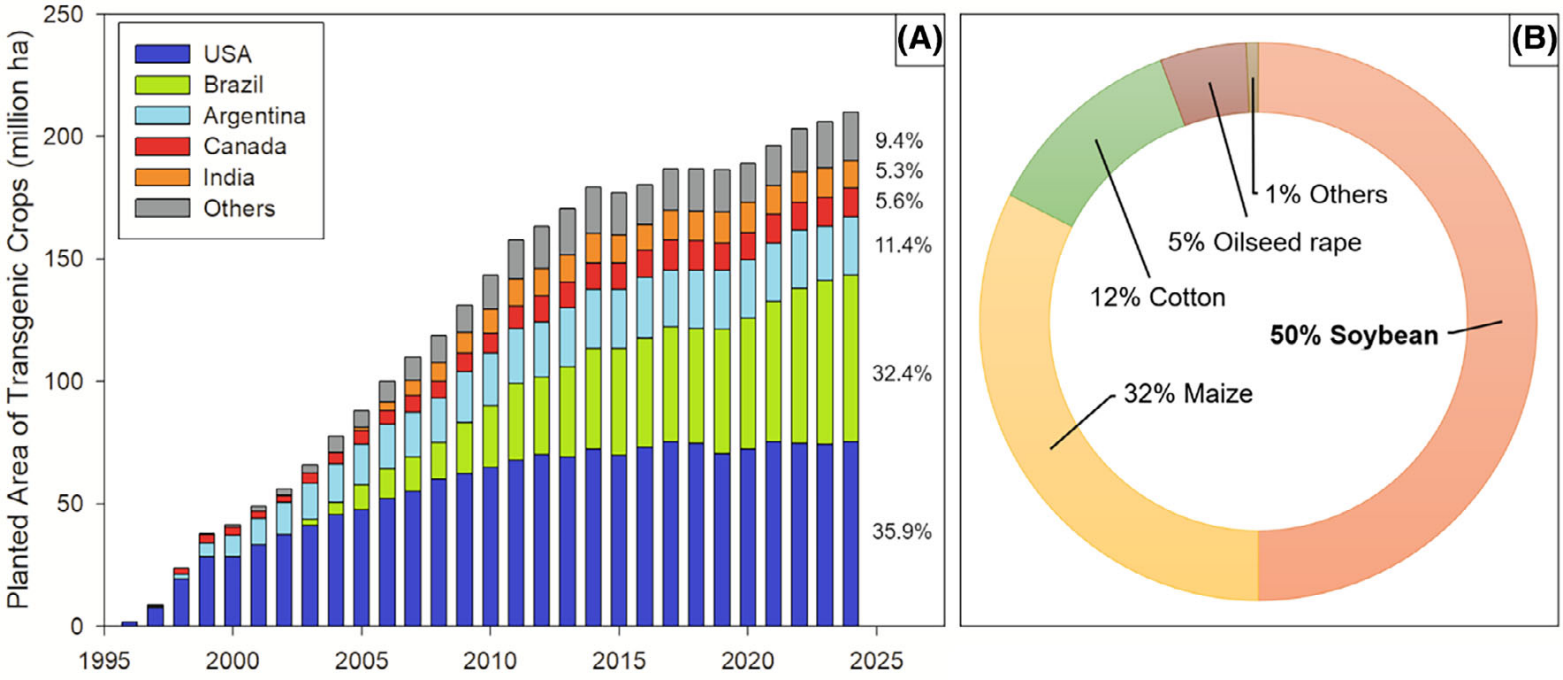
Trends in the global planting area of transgenic crops from 1996 to 2024 by country (A), and percentage of total GM‐planted area by crop in 2024 (B). Adapted from Agbio Investor.^12^

Because no agronomic crops are naturally tolerant to glyphosate, its use was initially restricted to non‐selective applications, effectively excluding major cropping systems from this highly effective herbicide. Thanks to its systemic action, broad‐spectrum control, relatively rapid environmental degradation, relatively low environmental impact, and over two decades of use without reported resistance, glyphosate became the ideal herbicide for developing transgenic, herbicide‐resistant crops, allowing its selective use in agronomic crops.^6^, ^11^ Glyphosate‐resistant (GR) crops were first commercialized United States (USA) in 1996, covering 1.7 million ha. By 2014, the global GR crop area had increased one hundredfold, reaching 179 million ha. However, from 2015 to 2024, this growth slowed considerably, rising to 209.8 million ha—a modest increase of 16.8% over 9 years (Fig. 1),^12^ partly due to market saturation. For example, close to 100% of the sugarbeet grown in the USA is GR, and GR soybeans, cotton, and maize were all around 90% in the USA by 2015.^6^ Similarly, Argentina began growing GR soybeans in 1996, with over 90% adoption by 2000.^13^ In Brazil, GR soybean was also the first transgenic crop to be approved for cultivation, in 2003,^14^ achieving adoption rates above 90% by 2014. Expansion of existing GR crop adoption continues to increase glyphosate use. Currently, 28 countries grow transgenic crops (most of which are GR), but over 80% of the global GR crop area is concentrated in the USA, Brazil, and Argentina (Fig. 1(A)).^13^ Although multiple transgenic technologies exist, providing resistance to insects, diseases, and other herbicides, most transgenic crops still include glyphosate resistance,^15^ often as part of stacked traits. The continued use of GR crops reflects the sustained agronomic value of glyphosate for weed control in these cropping systems and their relevance in plant breeding programs aimed at developing new transgenic cultivars.^16^ In practice, new herbicide‐resistance traits are commonly developed and deployed on GR events.^11^ Soybean, maize, cotton, and oilseed rape account for about 99% of all GR‐planted area (Fig. 1(B)).^12^ GR sugar beet and alfalfa are cultivated in the USA and Canada and GR eucalyptus and sugar cane are grown in Brazil.

The expiration of Monsanto's glyphosate patent in 2000 opened the global market to new manufacturers, lowering prices and expanding glyphosate's use,^3^ surpassing the use of any other herbicide mode of action (MoA).^2^, ^17^ By 2014, global agricultural use of glyphosate had reached 746 580 t (about 18% of all pesticides used worldwide), with 90% of this volume used in agricultural activities.^18^ In 2018, glyphosate was estimated to be used on approximately 477 million ha,^19^ representing 26% of the total planted area that year (1.87 billion ha). Glyphosate is widely used in conventional cropping systems (e.g., for pre‐plant weed control and pre‐harvest crop dessication), perennial crops, pastures, planted forests, and non‐agricultural areas such as roadsides and railways.^3^, ^18^, ^20^ In Brazil, glyphosate accounts for 36% of the pesticide market and 58% of the herbicide market, mainly due to the large areas planted with GR crops.^21^ However, even in countries with little or no GR crop cultivation, its use remains high. In Europe, for example, glyphosate accounts for about one‐third of total herbicide use and is applied on 30–50% of agricultural land, including annual and perennial crops, and infrastructure maintenance.^20^ Glyphosate usage in Europe varies by country, ranging from 15% in Turkey to 78% of herbicide usage in Greece.^18^ In Mexico, where GR crop area is limited, glyphosate still represents around 45% of the herbicide market, with a usage pattern similar to that in Europe.^22^

In the early years of its use, glyphosate was considered by some scientists to pose an extremely low risk for evolved resistance. This perception was based on several factors: the estimated low frequency of EPSPS resistance alleles (approximately 5 × 10^−8^), plants' limited ability to metabolize the compound, the absence of residual soil activity, and the assumed lower competitiveness of potential GR biotypes.^23^, ^24^ Furthermore, Monsanto assumed that more than one codon would have to mutate simultaneously to provide a robust, catalytically competent, resistant form of EPSPS, i.e., a form conferring high‐level resistance while maintaining sufficient enzymatic activity to sustain plant growth, the probability of which was considered to be almost infinitely low. To support this, mutagenesis studies with *Arabidopsis thaliana* resulted in no cases of glyphosate resistance, whereas chlorsulfuron and imazethapyr resistance occurred with a frequency of 3.2 × 10^−5^.^25^ Reliance on *A. thaliana* as model for all weeds was naïve.^26^ Furthermore, the experiment only showed that target‐site resistance (TSR) evolves faster for acetolactate synthase (ALS) inhibiting herbicides than for glyphosate,^25^ but it did not estimate the likelihood of non‐target site resistance (NTSR) evolving to glyphosate. The study also failed to recognize that robust resistance often results from the gradual accumulation of resistance alleles, a process termed ‘creeping resistance’,^27^ whereby multiple low‐effect mechanisms combine over time to produce high overall resistance levels. By the time the paper was published, glyphosate resistance had already been reported.^28^, ^29^ Although the first evolved GR weed was detected 22 years after glyphosate commercialization, supporting a slower evolution of resistance compared with some other herbicides, the rapid increase in GR weed species after 1996 showed that confidence in the predicted low risk of resistance evolution by some was unfounded.^29^, ^30^ The intensive and repeated use of glyphosate, particularly after the widespread adoption of GR crops, greatly increased selection pressure on weed populations ensuring that resistance would evolve and spread in multiple species and agronomic settings.^31^, ^32^, ^33^

**Figure 2:**
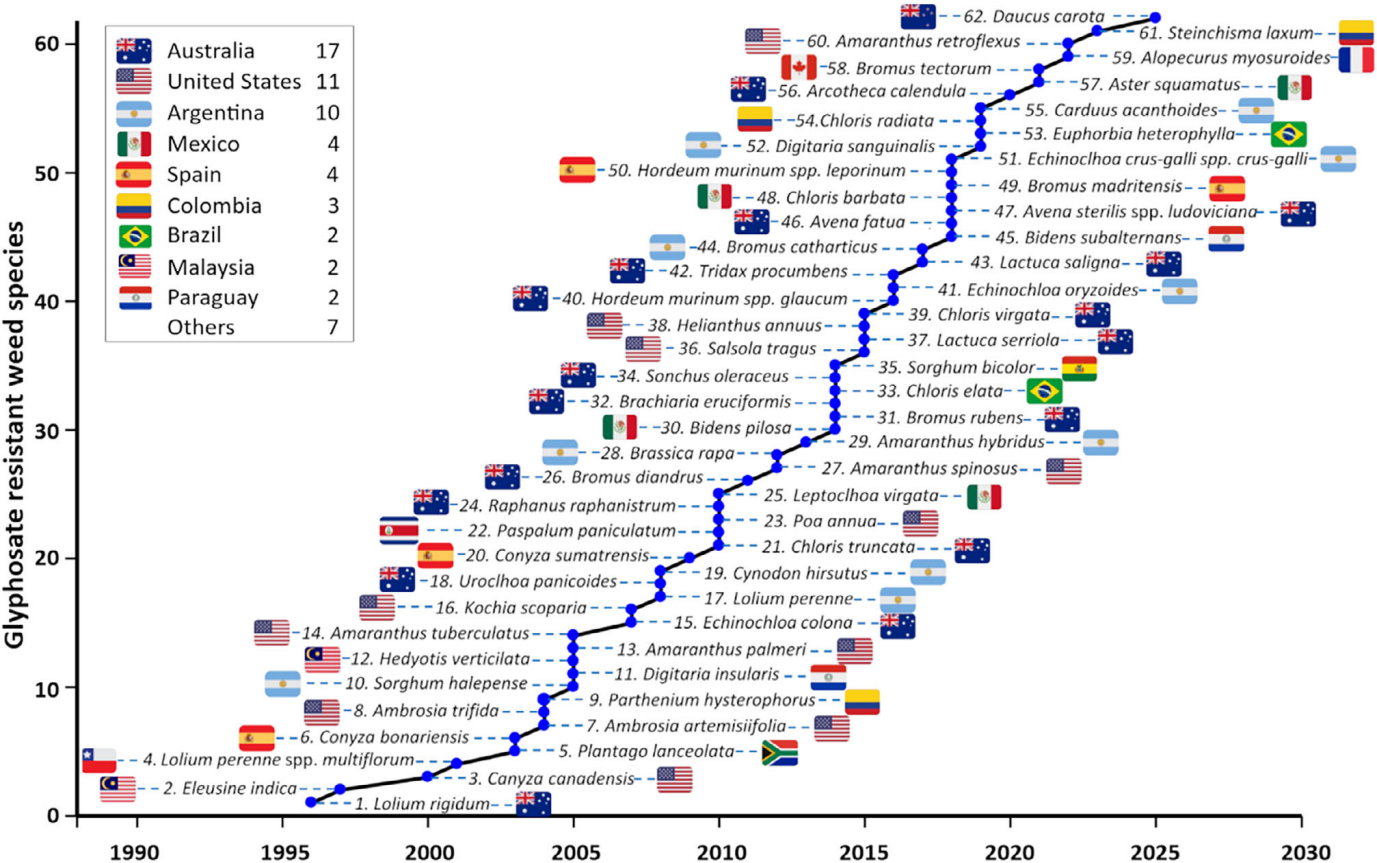
Chronological accumulation of first reports of unique cases of weed resistance to glyphosate in the world through September 26, 2025. Adapted from Heap.^34^

The first confirmed case of a glyphosate‐resistant weed was reported in 1996 in *Lolium rigidum* in Victoria, Australia,^28^ coincidentally and ironically, the same year that the first GR crop was introduced in the USA and that Monsanto published a paper suggesting that glyphosate resistance would not evolve.^23^ Since then, at least 62 weed species have evolved resistance to glyphosate across various crops and agricultural systems worldwide (Fig. 2).^34^ Evolved resistance to glyphosate is due to the widest range of known resistance mechanisms among herbicides,^35^ a reflection of both its extensive global use and the adaptive capacity of weed populations under strong and continuous selection pressure. An understanding of these mechanisms is useful for the development of sustainable weed management strategies such as herbicide rotation or tank mixtures with different MoAs and/or different chemistries, cultural practices, and mechanical weed control methods.^36^ Ignoring the need for diverse weed management strategies eventually undermined glyphosate's effectiveness.

Glyphosate and GR crops have played a major role in boosting global agricultural productivity, while also exerting substantial economic and environmental and societal impacts.^6^, ^37^, ^38^, ^39^ This review examines the global status of glyphosate resistance three decades after the first documented case in 1996, analyzing patterns of emergence and spread across weed species, crops, agronomic farming systems, and geographical regions, as well as resistance mechanisms. We also attempt to provide insights into future glyphosate and GR crops.

## 2 GLOBAL OVERVIEW OF GLYPHOSATE RESISTANCE

According to the *International Herbicide‐Resistant Weed Database* (IHRWD),^34^ glyphosate resistance has been documented in 62 weed species across 16 countries, considering unique first reports (species × herbicide) of resistance (Fig. 2). Australia leads with 17 first report cases, followed by the USA (11) and Argentina (10), which together account for 61% of all first global reports. Mexico and Spain each report four cases, followed by Colombia (3), and Brazil, Malaysia, and Paraguay (two each). Single cases have been documented in Chile, South Africa, Costa Rica, Bolivia, Canada, France, and New Zealand. The IHRWD does not capture all documented cases in the scientific literature. Consequently, additional GR weed species have been reported but are not yet included in the database, including *Amaranthus viridis* from Brazil,^40^ *Bromus sterilis* from England,^41^ *Chloris distichophylla* from Brazil,^42^ *Echinochloa chacoensis* from Argentina,^43^ and *Lamarckia aurea* from Mexico.^44^ Among the GR weed species included in the IHRWD, 33 are monocotyledonous (all within the Poaceae family, e.g., *Bromus spp*., *Chloris spp*., and *Lolium spp*.), while 29 are dicotyledonous distributed across multiple families. Asteraceae and Amaranthaceae are the most represented dicot families, mainly species of the genera *Conyza* and *Amaranthus*.

**Figure 3:**
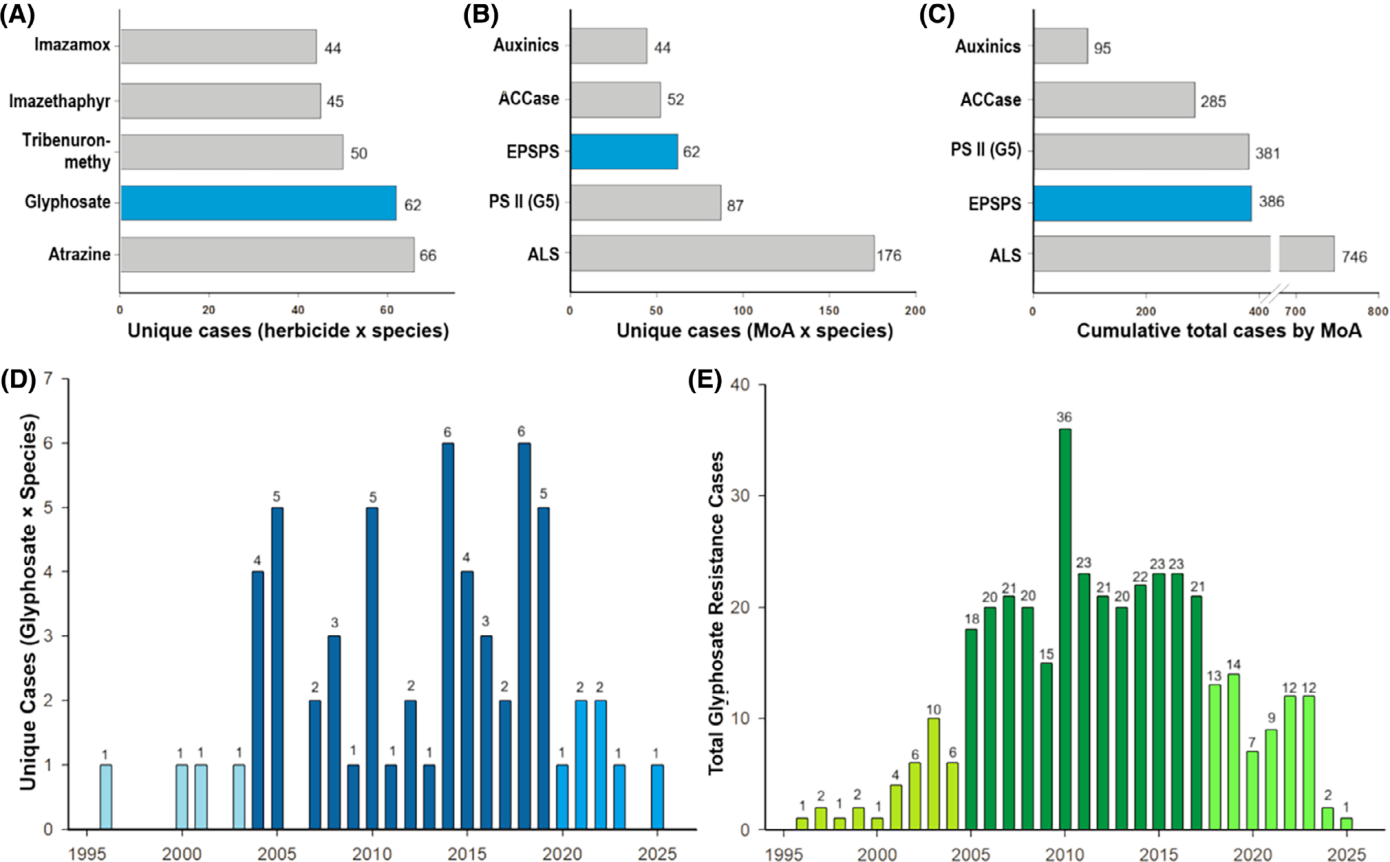
Overview of herbicide resistance cases by herbicide, mechanism of action (MoA), and glyphosate‐specific data through September 26, 2025. (A) Unique herbicide resistance cases by individual herbicide; (B) Unique resistance cases by MoA; (C) Cumulative total resistance cases by MoA; (D) Annual number of unique reported glyphosate resistance cases; and (E) Cumulative total of glyphosate resistance cases reported annually. Adapted from Heap.^34^

Glyphosate ranks second in the number of unique resistance cases, behind atrazine, which has 66 (Fig. 3(A)).^34^ Atrazine was first commercialized in 1959. When categorized by MoA, glyphosate ranks third in unique cases, and second in total cases (386 reports), including multiple occurrences of the same resistance in different regions. Acetolactate synthase (ALS) inhibitors lead both categories, with 176 unique and 746 total cases. Photosystem II (PSII – serine 264 binders, HRAC group 5) inhibitors rank second in unique cases with 87, and third in total cases with 381 (Fig. 3(B), (C)).^34^ Given its use over vast areas for decades, analyses show that glyphosate has a low to moderate rate of resistance evolution relative to the treated area compared with herbicides with other widely used MoAs, such as acetyl CoA carboxylase (ACCase), ALS, and PS II inhibitors.^45^

The temporal dynamics of GR reports show three phases (Fig. 3(D)).^34^ In the initial phase (1996–2003, 0.5 cases/year), cases were relatively few, perhaps due to limited detection capabilities and a lag phase in the evolution of GR weeds. From 2004 to 2019, cases increased markedly (3.1/year), with peaks in 2005, 2010, 2014, 2018 and 2019, reflecting widespread adoption of GR crops and greater selection pressure over large areas. Post‐2020, there was a decline or stabilization in unique cases (2020–2025, 1.2 cases/year). This trend does not indicate a reduction in the GR weed problem; rather, the slowing pace of new reports suggests that most weed species exposed to glyphosate for decades and capable of evolving resistance have already done so, leaving a diminishing pool of potential first reports despite continued selection pressure.

Regarding the total resistance cases (386), the early years (1996–2004) had low reports, averaging 3.7 cases per year. From 2005, there was a significant and consistent increase, remaining high until 2017, with an average of 23.3 cases per year and a peak in 2010 when 36 cases were reported (Fig. 3(E)).^34^ During this period, annual cases never fell below 15, highlighting a critical phase likely caused by wider resistance spread or control failures. After 2018, there has been a gradual decline in reports, averaging 8.8 cases per year between 2018 and 2025. This reduction may reflect a combination of factors, including advances in resistance management practices, changes in resistance monitoring intensity, and reduced interest in reporting additional cases due to limited novelty, particularly when new reports do not represent unique resistance cases.

**Figure 4:**
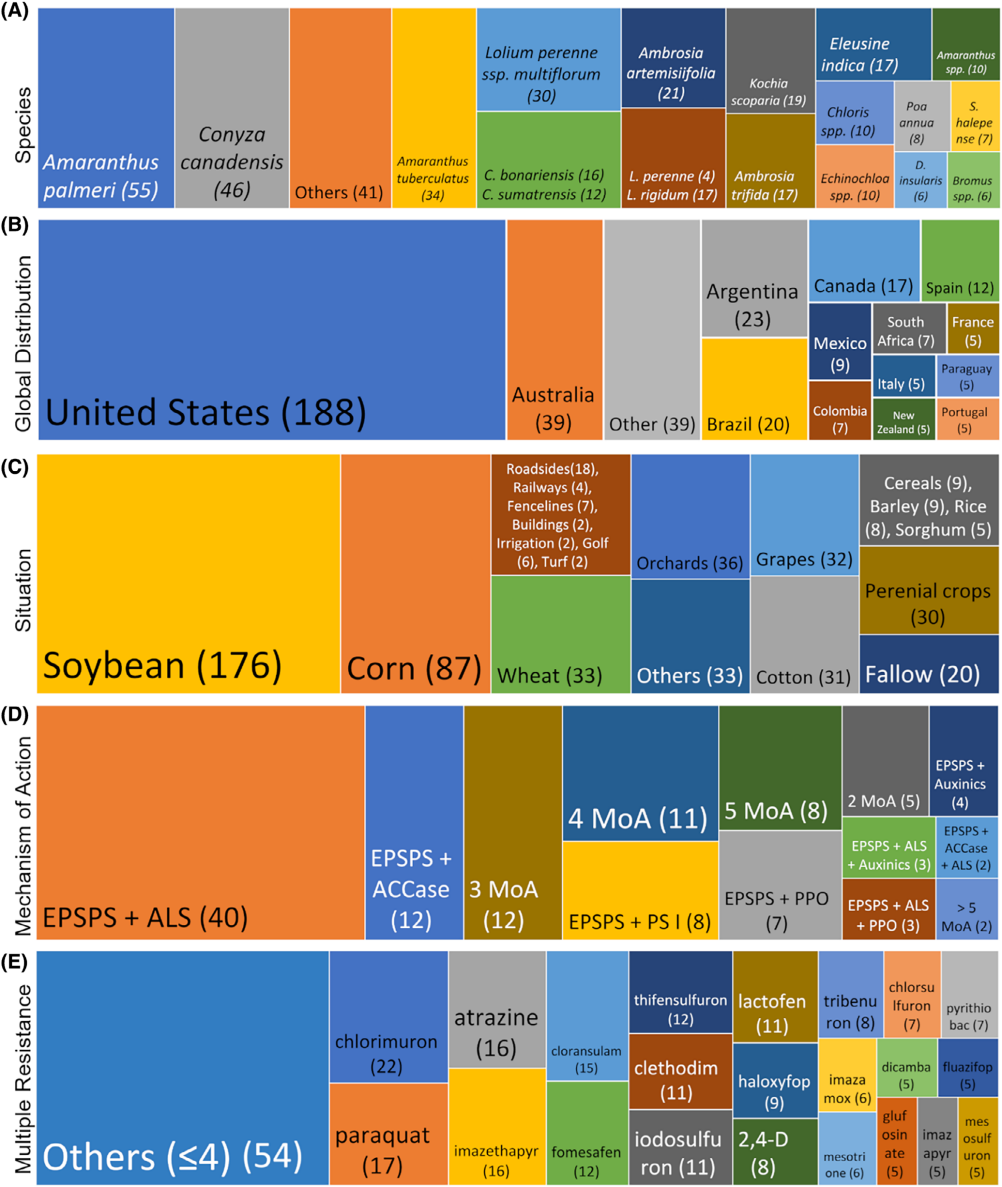
(A) Number of glyphosate resistance cases by weed species; (B) Geographic distribution and concentration of glyphosate resistance cases worldwide; (C) Contexts and cultivation settings where glyphosate resistance has been reported; (D) Mechanisms of action linked to glyphosate resistance; and (E) Most common herbicides associated with glyphosate resistance. The numbers in parentheses indicate the number of occurrences for each situation through September 26, 2025. Adapted from Heap.^34^

A small number of species account for the majority of reported GR cases, with *Amaranthus palmeri* accounting for 55 reports (14.3%), *Conyza canadensis* (11.9%), *Amaranthus tuberculatus* (8.8%), *Lolium perenne* ssp. *multiflorum* (7.8%), and *Conyza bonariensis* and *Conyza sumatrensis* together (7.3%). Also notable are *Ambrosia artemisiifolia* and *Lolium perenne* and *L. rigidum*, each with 5.4%, *Kochia scoparia* with 4.9%, and *Ambrosia trifida* and *Eleusine indica* with 4.4% each.^34^ Together, these 12 species represent 70% of all reported glyphosate resistance cases (Fig. 4(A)), highlighting their relevance and the need for targeted management strategies to control these highly adaptive, difficult‐to‐manage species with a high risk of herbicide resistance selection.

Glyphosate resistance has been reported in 31 countries,^34^ with the USA accounting for nearly half of the global total (49%), followed by Australia (10%), Argentina (6%), Brazil (5%), Canada (4%), and Spain (3%) (Fig. 4(B)). Together, these six countries account for 78% of all reported cases (299). This concentration reflects not only intensive glyphosate use and large‐scale agricultural systems, but also differences among countries in resistance detection capacity, monitoring intensity, publication practices, and reporting priorities. Consequently, some regions may be underrepresented despite evidence of resistance in the literature. For example, the IHRWD lists only two glyphosate resistance cases from China (*Conyza canadensis* and *Eleusine indica*), whereas additional cases have been documented in published studies (e.g., *Amaranthus palmeri*).^46^ Therefore, country‐level patterns derived from the IHRWD should be interpreted with caution and, where possible, complemented with information from the broader literature.

Glyphosate resistance has been reported in 557 situations, a number higher than the total number of cases reported because a single GR case may occur in multiple crops or environments.^34^ Soybean has the highest frequency, with 176 cases, followed by maize (87) and cotton (31). These three crops, largely GR in Brazil and the USA, account for 53% of all reported cases. Perennial crops such as olive, orange, oil palm, and almond (30 cases), along with other orchard crops (36) and vineyards (32), add up to 98 cases (18% of the total). Wheat, with 40 cases, accounts for 7%, while non‐agricultural areas, such as roadsides, railways, fences, buildings, irrigation systems, golf courses, and lawns, account for 41 cases (7%). These categories represent 32% of glyphosate resistance situations reported worldwide. Other cases include crops such as alfalfa, canola, and sugar beet (all three of which are GR crops grown in the USA) (33 cases), various cereals (31), and fallow areas (20), totaling 84 cases (15%) (Fig. 4(C)).^34^ These data indicate that the selection of GR weed biotypes occurs mainly in large‐scale crops and in environments with intensive glyphosate use. However, the high number of reports in perennial and non‐agricultural areas also reveals the importance of proper resistance management in these settings.

Of the 386 total GR cases, 269 refer exclusively to glyphosate resistance,^34^ while the remaining 117 involve multiple resistance, i.e., concurrent resistance to glyphosate and herbicides with other MoAs, including cross‐resistance. The most frequent combinations involving two MoAs are: EPSPS + ALS inhibitors (40 cases), followed by EPSPS + ACCase inhibitors (12), EPSPS + photosystem I (PS I) inhibitors (8), EPSPS + protoporphyrinogen oxidase (PPO) inhibitors (7), and EPSPS + auxinics (4). Common three‐way resistance combinations include: EPSPS + ALS + auxinics (3), EPSPS + ALS + PPO (3), and EPSPS + ACCase + ALS (2). There are also five and 12 cases of resistance to two and three MoAs, respectively, involving glyphosate combined with less common MoAs, such as: very long chain fatty acid synthase (VLCFAs) inhibitors, glutamine synthetase (GlS), PSII, hydroxyphenylpyruvate dioxygenase (HPPD), phytoene desaturase (PDS), microtubule assembly, and cellulose synthase inhibitors. Even more complex situations include resistance to four MoAs (11 cases), five MoAs (8), and more than five MoAs (2) (Fig. 4(D)).^34^ These data highlight the increasing complexity of resistance in weed species and reinforce the urgency of adopting integrated resistance management practices such as herbicide diversification using different MoAs, and the integration of cultural and preventive practices.

Glyphosate resistance has been reported in combination with 77 herbicides, totaling 315 mentions.^34^ The herbicides with 10 or more cases include chlorimuron (22), paraquat (17), atrazine (16), and imazethapyr (16). Others include cloransulam (15), fomesafen (12), thifensulfuron (12), clethodim, iodosulfuron, and lactofen, each with 11 mentions. These herbicides total 143 mentions (45% of the total). Among those with five to nine mentions are haloxyfop (9), 2,4‐D (8), tribenuron (8), chlorsulfuron (7), pyrithiobac (7), imazamox (6), and mesotrione (6). Other widely‐used herbicides such as dicamba, fluazifop, glufosinate, imazapyr, and mesosulfuron were cited five times each, totaling 76 mentions (24% of the total). The remaining 54 herbicides, such as metribuzin, pinoxaden, MCPA, nicosulfuron, diclofop‐methyl, diuron, oxyfluorfen, S‐metolachlor, and trifluralin, were mentioned up to four times each, totaling 96 mentions (31%) (Fig. 4(E)).^34^ These data reflect the growing complexity of cross‐ and multiple‐resistance.

Some cases of glyphosate resistance with multiple resistance to herbicides with other MoAs have been increasingly reported in different weed species, complicating chemical management. In Brazil, *C. sumatrensis* populations from Paraná have evolved resistance to five distinct MoAs: EPSPS, auxinics, PPO, PS II, and PS I. In the USA, an *A. palmeri* population from Kansas has evolved resistance to six MoAs, including ALS, EPSPS, auxinics, PPO, PS II, and HPPD. *Lolium rigidum* from South Australia has evolved resistance to ACCase, ALS, EPSPS, PS II, and PS I herbicides. *Poa annua* from Tennessee, USA, represents one of the most complex cases, with resistance to seven MoAS: ALS, EPSPS, auxinics, PPO, PS II, GlS, microtubule assembly, and cellulose synthase. Besides glyphosate, the herbicides reported in these resistance cases include 2,4‐D, paraquat, glufosinate, atrazine, diuron, clethodim, chlorsulfuron, fomesafen, saflufenacil, mesotrione, metribuzin, simazine, indaziflam, and prodiamine.^34^

The above information from the IHRWD may not accurately reflect the actual situation in each country, as reporting criteria vary widely. Although the USA appears to be the most affected country and glyphosate resistance may seem closely linked to the adoption of GR crops, the database also reflects differences in investment in research, surveillance, and systematic reporting. For example, the USA (in total cases) and Australia (in unique cases) stand out due to strong research infrastructure and well‐established monitoring systems, which directly contribute to higher reporting rates.^34^ In contrast, Brazil typically reports only the first occurrence of resistance in the IHRWD. For example, GR *Digitaria insularis* was first reported in 2008, followed by multiple resistance to glyphosate + ACCase inhibitors in 2020.^34^ As a result, only two entries appear in the IHRWD, despite national surveys documenting more than 2500 GR populations across the country,^47^ with estimates suggesting that about 25% of Brazil's agricultural land has some degree of infestation with *D. insularis* populations resistant to glyphosate and ACCase inhibitors.^48^ Many GR weeds have been reported in Spain, despite the absence of GR crops. This may be largely due to extensive research on GR weeds led by the University of Córdoba across the Iberian Peninsula.^49^ In Mexico, most glyphosate resistance cases (7 out of 9) come from citrus orchards in the Gulf of Mexico and Pacific coast,^50^ and were documented by a single research group. This pattern does not imply the absence of resistance in other crops and regions, but rather reflects limited national capacity for comprehensive resistance surveys.^22^, ^50^

To some extent, the number of GR weed cases reported in the IHRWD reflects the extent of glyphosate use. However, their uneven distribution across countries and cropping systems indicates that reported cases are also strongly influenced by each country's technical and institutional capacity to detect and report resistance, rather than by the true magnitude of the problem. An important source of bias in the IHRWD is the treatment of geographic units: the USA, Canada, and Australia are reported at the state or provincial level, inflating apparent case numbers, whereas other major agricultural countries (e.g., Russia, China, Brazil, India, and Argentina) are reported as single entities despite comparable or larger land areas. Metrics such as total infested area would be more informative, but such estimates can be highly uncertain. Accurately quantifying the area infested by GR weeds is inherently difficult in any country, as cases vary from small, localized patches to extensive infestations across production regions and are rarely assessed using standardized methods. As a result, reported resistance cases should be interpreted primarily as indicators of presence rather than precise measures of spatial extent or impact. The economic impacts of GR weeds are discussed in Section 5.

Although the increases in GR weeds (in terms of both species and geographical locations) detailed by the IHRWD cannot provide a fully accurate assessment of their agricultural impact, these data clearly demonstrate that GR weeds have become a major problem. The need for and commercial success of stacking resistance traits to additional herbicides in GR crops provides strong evidence that reliance on glyphosate alone is no longer a viable management strategy in GR cropping systems.^51^ However, the use of glyphosate in the USA had not been reduced in 2019 (the last year of the USGS pesticide use data) from when its use plateaued in 2011,^52^ despite the widespread adoption of stacked herbicide resistance trait cultivars. During the same period (2011–2019) the use of other selective postemergence herbicides (e.g., acifluorfen and lactofen) in soybeans increased in Brazil.^45^, ^52^ Thus, strong selection pressure from glyphosate continues, selecting for new GR weeds species and ensuring that existing GR weed species continue to thrive, even if there are fitness costs associated with some of the resistance mechanisms.

## 3 GLYPHOSATE RESISTANCE MECHANISMS

**Figure 5:**
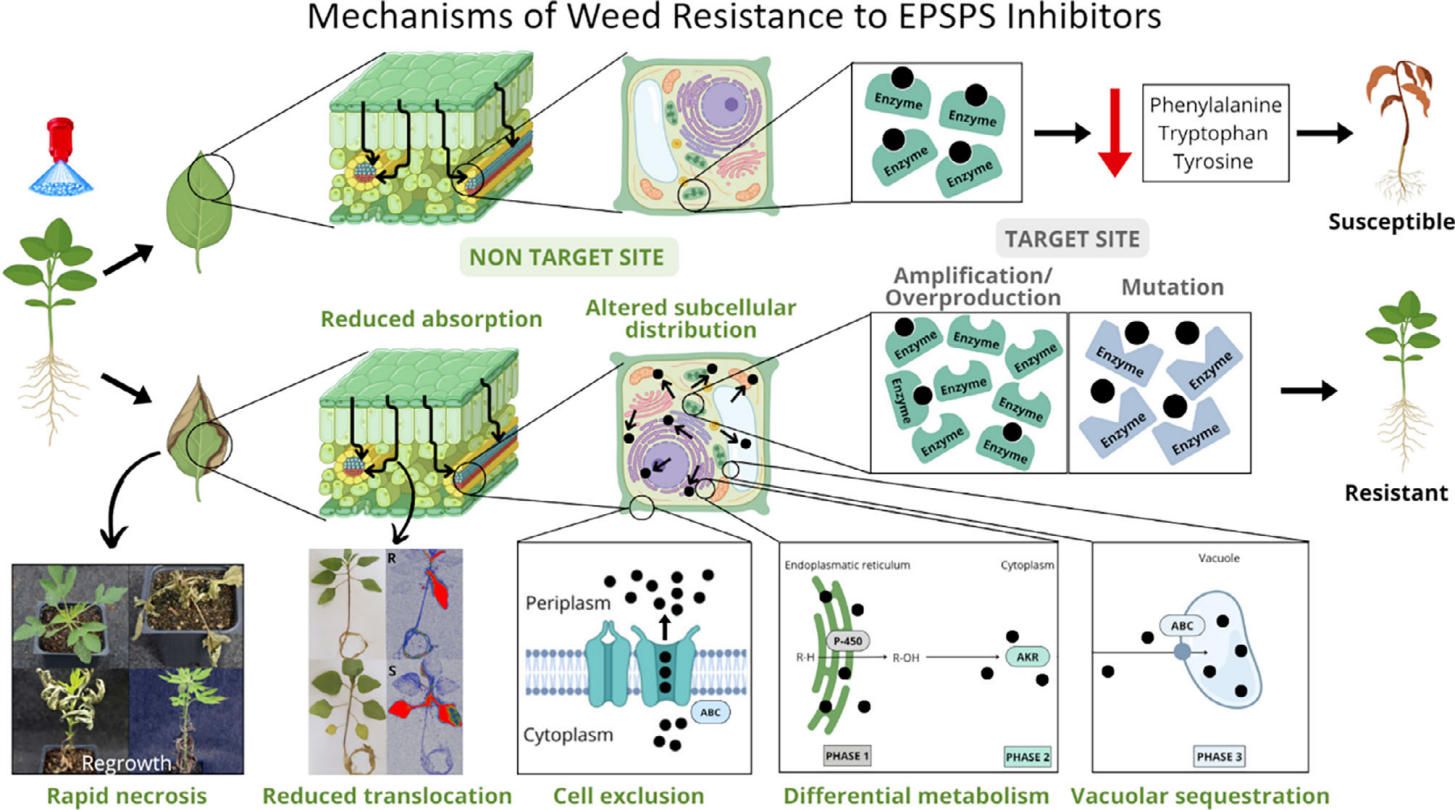
Route of glyphosate until it reaches the target site in a susceptible plant and the expression of target site (TSR) and non‐target site (NTSR) resistance mechanisms in a resistant plant.

Glyphosate resistance involves TSR mechanisms, which include molecular alterations in the EPSPS enzyme or increased EPSPS abundance, and NTSR mechanisms, encompassing anatomical, physiological, and metabolic processes that reduce the amount of glyphosate reaching EPSPS.^53^ In many GR weeds, both TSR and NTSR mechanisms coexist. The gradual accumulation of multiple resistance mechanisms over time (creeping resistance) reflects the fact that no single mechanism alone, other than gene amplification, provides sufficient resistance to withstand the high glyphosate doses sometimes applied. Although a large number of GR weed species exist, this does not explain the greater diversity of resistance mechanisms for glyphosate than for any other herbicide (Fig. 5). There are other MoA herbicide classes for which more resistant weed species have been reported (Fig. 2), but all have fewer resistance mechanisms.

### 3.1 Non‐target‐site resistance – NTSR

NTSR mechanisms are typically regulated by multiple genes unrelated to the molecular target of a herbicide, each contributing incrementally to resistance, which can make their characterization complex.^54^ There have been more NTSR mechanisms for glyphosate than for any other herbicide.^55^

#### 3.1.1 Reduced foliar absorption

Compared to most herbicides, glyphosate enters living cells slowly because of its high hydrophilicity.^3^ After crossing the leaf cuticle, glyphosate entry into cells, including phloem cells, is partly mediated by phosphate transporters located in the cell membrane.^56^ Reduced loading of glyphosate onto these transporters in GR plants, due to limited penetration or altered transporter activity, represents a NTSR mechanism that generally confers low resistance levels.^54^, ^56^ Leaf surface traits, including cuticle thickness and composition as well as trichome presence, density, and morphology, can further influence glyphosate penetration.^57^, ^58^, ^59^

Reduced absorption of glyphosate in GR biotypes has been documented in several weed species. For example, GR *Chloris elata* biotypes from Brazil, resisted glyphosate partly through reduced absorption.^60^ The glyphosate susceptible (GS) biotype absorbed 12% more of the applied ^14^C‐glyphosate compared with the GR biotype. Only a NTSR mechanism was confirmed by the absence of mutations in the Pro106 codon of the *EPSPS* gene and no differences in *EPSPS* copy number or gene expression between the R and S biotypes.

#### 3.1.2 Reduced translocation

For systemic herbicides such as glyphosate, translocation from the application site to plant meristems is crucial.^3^, ^56^ Reduced translocation is a well‐documented NTSR mechanism associated with glyphosate resistance. The exact mechanisms responsible for reduced translocation are not yet fully understood, partly because it is usually assessed using radiometric methods with ^14^C or ^3^H‐labeled glyphosate. These methods have limitations, as they cannot distinguish between herbicide in the apoplastic and symplastic compartments, nor do they identify possible metabolites.^61^, ^62^ However, reduction in glyphosate translocation has been associated with changes in subcellular distribution or vacuolar sequestration, mechanisms that may involve active tonoplast transporters or ATP‐binding cassette (ABC) transporters, which are described below.

In some GR weed biotypes, glyphosate transport within the treated leaf occurs predominantly via the transpiration stream rather than through the phloem. As a result, the herbicide accumulates in the distal portion of the leaf through xylem‐mediated movement, thereby limiting its redistribution to meristematic tissues.^58^

Reduced translocation in GR biotypes of *C. canadensis* and *L. rigidum* conferred levels of resistance three to four times higher than that observed in the GR biotype of *Eleusine indica* with the Pro106Ser mutation in the *EPSPS* gene.^63^, ^64^, ^65^ In populations of *Sorghum halepense* in Argentina, GS plants translocated 26 to 29% of ^14^C‐glyphosate to the root and stem meristems, while in GR plants translocation was reduced to 9 to 11%.^66^ Glyphosate resistance of Australian *L. rigidum* was associated with changes in translocation.^67^ This GR biotype translocated less ^14^C‐glyphosate to the meristems, especially to those of the stem. In the case of *C. eleta* mentioned before,^60^ in addition to having reduced glyphosate uptake, translocation of glyphosate taken up was only 27% in the GR biotype, compared to 36% for the GS biotype.

In GR *A. palmeri* populations from Argentina, there was low absorption and limited translocation of glyphosate.^59^ Compared with a GS population, GR plants absorbed around 10% less ^14^C‐glyphosate and translocated about 20% less of the herbicide to the shoots and roots, retaining 62% of the ^14^C‐glyphosate in the treated leaves 96 h after treatment. Previous reports of glyphosate resistance in *A. palmeri* were related to a TSR mechanism, mainly gene amplification (see below).^35^ Although populations from Argentina and Mexico were reported to possess a mutation (Pro106Ser) and NTSR mechanism,^68^, ^69^ this was the first known case of glyphosate resistance in *A. palmeri* apparently mediated exclusively by a NTSR mechanism.^59^

#### 3.1.3 Vacuolar sequestration to prevent translocation and movement to EPSPS

Rapid vacuolar sequestration of glyphosate can reduce translocation. Furthermore, this process in any cell type reduces the amount of herbicide in the cytoplasm, preventing it from reaching the chloroplasts and other plastid (e.g., leucoplasts and etioplasts), where EPSPS is located. Plastid‐located EPSPS is an essential enzyme in all cell types, whether photosynthetic or not. Glyphosate can also bind nuclear‐coded, but cytoplasm‐translated EPSPS in the cytoplasm before it enters the plastid, preventing its entry into the plastid.^70^ How much of glyphosate's effect is due to this process, *versus* inhibition of the catalytic activity of EPSPS in the plastid is unknown, but vacuolar sequestration prevents both processes. This sequestration is mediated by active membrane transporters present in the tonoplast, mainly ABC transporters,^71^, ^72^, ^73^ which hydrolyze ATP to promote the transport of glyphosate into the vacuole. This mechanism restricts systemic movement, especially to root and shoot apical meristems, by confining glyphosate near the site of absorption.^74^

Vacuolar sequestration of glyphosate labeled with ^31^P was characterized using a nuclear magnetic resonance (NMR) technique.^74^, ^75^ Based on the pH differences between the cytoplasm and the vacuole, the concentration of glyphosate was determined in the vacuoles of GR and GS biotypes of *C. canadensis*.^74^ In these plants, up to 85% of the applied glyphosate was sequestered in the vacuoles 4 h after treatment, in contrast to only 15% in GS plants. Subsequently, this NTSR mechanism was reported in populations of GR *Lolium spp*. from different countries.^76^

Vacuolar sequestration of glyphosate is regulated by an oxygen‐dependent active transport mechanism. Decreased oxygen levels reduce the availability of ATP, which in turn limits the transport of the herbicide to the vacuoles.^54^ In addition, environmental factors, especially temperature, also influence this mechanism. The expression of genes encoding ABC transporters increases at relatively high temperatures, thereby favoring vacuolar sequestration. Conversely, at lower temperatures, the expression of these genes is reduced, decreasing the activity of the transporters and allowing more herbicide to be translocated.^73^, ^76^ This temperature‐dependent effect has been observed in *C. sumatrensis*, *L. multiflorum* and *S. halepense*.^77^ The expression of ABC transporters seems to be coordinated with the expression of the *EPSPS* gene. At lower temperatures, the expression of the *EPSPS* gene is also reduced, limiting the amount of the enzyme to be inhibited.^73^, ^78^ Therefore, a lower concentration of glyphosate is enough to inhibit sufficient EPSPS to be herbicidal, which, under field conditions, translates into improved effectiveness of lower herbicide doses on cooler days or in more temperate climates.

The resistance of a GR *C. bonariensis* biotype was not due to reduced uptake, translocation, or altered EPSPS;^79^ however, there was much reduced shikimate accumulation in sink tissues (roots and young leaves) to which glyphosate was translocated in the GR biotype compared to the GS biotype. A possible explanation is that glyphosate failed to reach the plastids where EPSPS resides, possibly due to vacuolar sequestration. Glyphosate resistance has also been proposed to result from altered transport of glyphosate into the plastid.^33^ To date, there is no proof of this mechanism of resistance to glyphosate.

#### 3.1.4 Cell exclusion to prevent translocation and movement to EPSPS

Cell exclusion can reduce intracellular glyphosate concentrations by pumping the molecule from the cytoplasm to the apoplast, limiting its access to EPSPS. This mechanism was identified in a GR biotype of *Echinochloa colona*, which overexpressed a C‐type ABC transporter EcABCC8, located in the plasma membrane.^80^ This transporter acts as an efflux pump exporting glyphosate from the cytoplasm to the apoplast, reducing its intracellular concentration.^71^ In transgenic rice plants overexpressing this gene, a significant increase in the rate of glyphosate efflux into the apoplast was found.^80^ Despite this resistance, no differences in foliar absorption or whole‐plant translocation of ^14^C‐glyphosate were detected between GR and GS rice plants,^80^ likely reflecting limitations of radiometric approaches.^62^, ^63^ Overexpression of the *EcABCC8* gene may be associated with epigenetic modifications. GR plants showed reduced DNA methylation in promoter regions (CHH motifs) and increased methylation within coding regions (CG motifs), consistent with epigenetic regulation of gene expression that may be responsible for maintaining the high basal expression of the EcABCC8 transporter under varying environmental conditions.^80^

The role of ABC transporters as mediators of resistance to xenobiotics is well documented in human cells. ABCC transporters (ATP‐binding cassette subfamily C) are ATP‐driven efflux pumps that couple nucleotide binding and hydrolysis to the transmembrane transport of structurally diverse endogenous compounds and xenobiotics, and are widely implicated in cellular detoxification and multidrug resistance mechanisms.^81^ For example, the HsABCC1 (MRP1) transporter confers drug resistance in tumor cells by exporting chemotherapeutic agents from the cytoplasm,^82^ using a mechanism analogous to that observed in *E. colona*. This analogy between such distantly related organisms highlights the evolutionary relevance of the cell exclusion mechanism as a resistance mechanism. Thus, the identification of the EcABCC8 transporter involved in glyphosate resistance demonstrates the potential role of membrane proteins in mitigating herbicide toxicity. Consistent with this broader relevance, glyphosate has been shown to increase ABCC transporter activity in the gills, but not in the brain, of zebrafish (*Danio rerio*)^83^ suggesting that glyphosate can interact with or influence ABCC transporter activity across taxa. Importantly, any direct interaction between glyphosate and plant ABC transporters does not appear to be detrimental, as crops with a GR EPSPS transgene have resistance factors of 50‐ to 100‐fold. However, further research is needed to determine the extent of involvement of ABC transporters in glyphosate resistance in other GR weeds.

#### 3.1.5 Rapid necrosis to prevent translocation to meristems

Rapid leaf necrosis, also known as the hypersensitive reaction or ‘phoenix phenomenon’, limits glyphosate translocation from treated leaves to metabolic sinks such as younger leaves and meristems, allowing plants to survive the herbicide application.^84^ Unlike vacuolar sequestration, this mechanism works by quickly killing treated leaf cells, thereby preventing glyphosate translocation beyond the application site. This symptom is similar to the mode of action of extremely fast‐acting, contact herbicides like paraquat, which fail to control regrowth because treated foliar tissues die before sufficient herbicide can be translocated to protected younger tissues and meristems. The appearance of this mechanism for glyphosate resistance was unexpected because glyphosate is normally a slow‐acting herbicide, even at high concentrations.

Rapid necrosis was identified in GR biotypes of *Ambrosia trifida* and *Aster squamathus*.^85^, ^86^ In these species, the herbicide was absorbed normally, but its translocation was significantly reduced due to rapid necrosis of treated foliar tissues. GR plants with this NTSR mechanism develop localized leaf necrosis within a few hours after application. Older sprayed leaves suffer complete death and desiccation within 6 h, followed by abscission within 24 h, while young and meristematic tissues not in contact with spray droplets remain less affected, allowing the plant to regrow from axillary buds and apical meristems a few days after application.

Although the mechanism of rapid necrosis induced by glyphosate is not yet fully understood, advances have been made in elucidating its physiological basis. Reactive oxygen species (ROS) accumulate rapidly after application of glyphosate, especially in older leaves.^84^, ^86^ The partial alleviation of necrotic symptoms by exogenous aromatic amino acids suggests an association with EPSPS inhibition and metabolic imbalances in the shikimate pathway. However, ROS production is generally considered a secondary stress response rather than a direct effect of glyphosate action.^3^, ^87^ In GS sugarbeet (*Beta vulgaris*), glyphosate causes unusually rapid physiological effects, including variable fluorescence indicators associated with inhibited carbon fixation.^88^, ^89^ This response may reflect a disruption of carbon allocation following aromatic amino acid depletion, potentially diverting carbon toward shikimate synthesis. Under high light conditions, impaired carbon fixation could cause ROS formation due to uncoupling of photosynthetic electron transport from carbon assimilation. However, the mechanistic link between EPSPS inhibition and accelerated ROS accumulation remains unresolved.

#### 3.1.6 Enhanced metabolic degradation

The metabolic degradation of most herbicides is a complex process involving the action of various detoxifying enzymes and typically consists of activation (phase I) and/or conjugation (phase II).^90^ The major enzyme systems responsible for herbicide detoxification, including cytochrome P450 monooxygenases (CYP450), glutathione‐transferases, and glycosyl transferases, are generally not involved in glyphosate degradation.^91^ This likely reflects the distinct physicochemical properties of glyphosate compared with most other herbicides (other than glufosinate, paraquat, and diquat), especially those that are oxidized by CYP450 enzymes.^92^

Most plants are capable of metabolizing most herbicides to some degree, but the difference between susceptibility and resistance often depends on the rate at which this metabolism occurs. Faster herbicide metabolism in crops, relative to the much slower metabolism in weeds, sustains the selectivity of many herbicides. Metabolic resistance arises when weed biotypes that were previously unable to detoxify a herbicide efficiently evolve enhanced metabolic capacity. Enhanced herbicide metabolism is currently a major challenge in managing resistant weeds, as it is often associated with cross‐ and multiple‐resistance to herbicides with different MoAs.^93^

Most, if not all, plant species are capable of metabolizing glyphosate to some extent, although typically at rates insufficient to confer resistance.^55^, ^94^, ^95^ This general metabolic capacity does not imply resistance, but rather reflects intrinsic detoxification processes present in plants. For weed species that metabolize glyphosate, one would expect selection pressure to select for enhanced degradation, but this has rarely been found to be the case. This is unexpected, considering the strong selection pressure exerted by the herbicide and its slow action, which theoretically provides time for its metabolic degradation to non‐lethal levels.^55^, ^91^ Most research suggests that glyphosate metabolism in plants is mediated by enzymes similar to glyphosate oxidoreductase (GOX), found in microorganisms, which convert glyphosate to aminomethylphosphonic acid (AMPA) and glyoxylate.^91^, ^94^ AMPA is the most commonly found glyphosate metabolite in glyphosate‐treated plants.^95^

Another degradation pathway involves C‐P lyase, commonly found in soil microbes, that converts glyphosate to sarcosine and inorganic phosphate. Sarcosine is the second most common glyphosate metabolite found in plants; however, researchers look for it less often, and it may have a shorter *in vivo* half‐life than AMPA, making this means of degradation more difficult to detect. Interpretation of glyphosate metabolism in plants is complicated by the potential involvement of endophytic microorganisms, as microbial glyphosate degradation is widespread and some endophytes (e.g., *Enterobacter cloacae*) are capable of metabolizing glyphosate.^96^ Endophytic degradation of other herbicides has been associated with natural herbicide tolerance,^97^ suggesting a possible, though still poorly understood, contribution to glyphosate metabolism in plants.

Some GR weed biotypes accumulate more AMPA than GS biotypes, but researchers have been skeptical because higher activity of a GOX‐type enzyme was not proven.^91^ The first well‐documented case of differential metabolism supported by enzyme‐level evidence was *E. colona*.^98^ In this species, resistance was attributed to overexpression of the aldo‐keto reductase (AKR) enzymes EcAKR4‐1 and EcAKR4‐2 (designated as AKR4C16 and AKR4C17), which can oxidize glyphosate to AMPA and glyoxylate using NADP^+^ as a cofactor.^99^ Although AMPA levels in the GS and GR biotypes were not measured after glyphosate treatment,^98^ and the broad substrate specificity of AKRs initially raised doubts about their primary role in resistance,^55^, ^100^ subsequent studies demonstrated the involvement of AKR family enzymes in glyphosate metabolism through gene silencing and mutational analyses.^101^ Structural and engineering analyses of these enzymes corroborated metabolic resistance in *E. colona*.^102^ Thus, some, if not all, of the earlier claims of elevated metabolic degradation of glyphosate in GR biotypes may be valid.

Environmental conditions influence the activity of AKR enzymes. GR *E. colona* plants grown at 35/30 °C survived the application of 540 g a.e. ha^−1^, producing 95% more biomass than the untreated control. In contrast, 30% of GR plants grown at 20/25 °C died, and the biomass of the survivors was reduced by 70%.^98^ Thus, the AKRs in this biotype appear to be more efficient at high temperatures, although neither glyphosate nor its metabolites were measured in this experiment.

Transcriptomic, metabolomic, and transgenic approaches have made it possible to identify other AKR genes linked to glyphosate resistance in other weeds. In *L. rigidum* expression of the *LrAKR4C10* and *LrAKR1* genes increased 4.3 times after glyphosate application. In addition, *Escherichia coli* cells transformed with these genes became resistant to glyphosate, and transgenic rice seedlings overexpressing the *LrAKR* genes survived high doses of the herbicide.^103^ In two GR biotypes of *E. indica*, up‐regulation of the *AKR4C10* gene was identified using RNA‐Seq, and for the first time, up‐regulation of the *CYP88* gene after glyphosate application was also identified.^104^

Xenobiotic chemicals often upregulate genes encoding detoxification enzymes, whether the enzymes that they encode can use the chemical as a substrate or not.^105^ For glyphosate, the involvement of CYP450s in metabolism had long been considered unlikely; however, recent studies suggest a possible contribution of specific CYP450s to glyphosate resistance in some weed species. In GR *E. indica* populations, upregulation of the CYP71AK44 gene was observed, and treatment with a CYP450 inhibitor reduced resistance levels from 8.5‐ to 3.6‐fold.^106^ Similarly, GR *Polypogon fugax* populations overexpressed *PfCYP51*, which conferred increased glyphosate resistance when inserted in yeast.^107^ However, these studies did not compare glyphosate metabolites in glyphosate‐treated GS and GR biotypes, nor did they demonstrate that glyphosate can be a substrate for the CYP450 by *in vitro* enzymology. If CYP450 enzymes are involved in glyphosate metabolism, one would expect such a resistance mechanism to be common, as it is for many other herbicide classes in weeds and for herbicide tolerances in crops. Furthermore, glyphosate itself has been reported to inhibit at least one plant CYP450 enzyme,^108^ although such an effect is unlikely to be associated with the MoA of glyphosate, given the high resistance factors (50–100‐fold) conferred by single transgenes encoding glyphosate‐resistant EPSPS in crops.^109^, ^110^

In principle, glyphosate resistance based on enzymatic degradation could result from changes in expression of a single gene or modification of a single enzyme. A GR crop that owes its resistance to metabolic degradation would be highly desirable, as it would have low levels of glyphosate residues in the edible product. However, resistance based on enhanced metabolic degradation remains rare in both natural weed populations and GR crops. In the case of industry, a GOX gene from *Ochrobactrum anthropi* was used in the first GR oilseed rape varieties, along with a transgene (*CP4*) for a microbial GR EPSPS, although later varieties had only *CP4*.^11^ Why the GOX gene was used in the first place and why it was later dropped was never revealed. More recently, GR oilseed rape with a microbial glyphosate acetyl transferase (GAT) transgene was made available, following the discovery and directed evolution of a glyphosate tolerance gene capable of acetylating glyphosate and thereby reducing its phytotoxicity.^111^

Why are there so few cases of glyphosate resistance based on enhanced degradation, especially given that plants appear to possess enzymes capable of degrading this herbicide? This question is even more perplexing considering the relatively slow mode of action of glyphosate. A plausible explanation is that plant enzyme(s) capable of degrading glyphosate have evolved to fulfill essential metabolic functions, and that altering these enzymes to enhance glyphosate metabolism, or overexpressing them, could disrupt their essential roles.

### 3.2 Target‐site resistance – TSR

TSR resistance is typically monogenic and involves alterations in the gene encoding EPSPS or increasing the amount of endogenous EPSPS, allowing GR plants to maintain metabolic pathways that glyphosate would otherwise disrupt, even when the herbicide reaches levels that would normally kill GS plants.^112^

#### 3.2.1 Mutations of the *EPSPS* gene

Mutations of *EPSPS* genes are the most common reported mechanism of resistance, despite the predictions that they were unlikely to occur.^23^, ^24^ Table 1, ^142^ summarizes the wide range of EPSPS mutations documented under field conditions. These mutations originate from pre‐existing or newly occurring codon changes that result in amino acid substitutions in the EPSPS protein.^35^ To successfully impart robust resistance, the mutation must structurally alter the EPSPS glyphosate binding site, without altering its function or catalytic activity to a point that would compromise the plant's survival in the absence of glyphosate.^35^, ^53^ Consequently, resistance‐conferring mutations are typically located in conserved regions or at key residues near the herbicide‐binding site. In the *EPSPS* gene, most resistance‐associated mutations are clustered between positions 95 and 107 (^95^LFLGNAGTAMRPL^107^),^143^, ^144^ which form a critical region for glyphosate interaction. However, mutations outside this core region have also been associated with resistance, such as Val133Ile and Pro382Leu in *E. indica*, Asp71Met, Ala112Ile, and Val201Met in *Ophiopogon japonicus, Liriope platyphylla*, and *L. spicata*, Glu91Ala in *Chloris truncata*,^145^, ^146^, ^147^ among others.

**Table 1:** First occurrence of major mutations in the EPSPS gene associated with glyphosate resistance in different weed species

Mutation	Species	Year	Country	GR_50_ S^†^	FR^‡^	RL^§^	Ref.
Pro‐106‐Ser	*Eleusine indica*	2002	Malaysia	–	2–4	r	^63^
	*Lolium perenne* ssp. *multiflorum*	2007	United States	60	2–5	r	^113^
	*Lolium rigidum*	2012	Australia	88–94	6–11	r/R	^114^
	*Amaranthus tuberculatus*	2013	United States	280	5	r	^115^
	*Echinochloa colona*	2013	United States	140	6.6	r	^116^
	*Lolium perenne*	2015	New Zealand	200	7–25	r/R	^117^
	*Parthenium hysterophorus*	2016	Dominican Republic	47	5.4–20	r/R	^118^
	*Leptochloa virgata*	2016	Mexico	138	3.3–5.2	r	^119^
	*Chloris virgata*	2018	Australia	515–594	2–9.7	r	^120^
	*Conyza canadensis*	2018	Canada	134	–	–	^121^
	*Chloris elata*	2017	Cuba	88	15	R	^122^
	*Amaranthus palmeri*	2017	Mexico	16	12–83	R	^68^
	*Chloris barbata*	2018	Mexico	127	12–15	R	^123^
	*Chloris radiata*	2021	Colombia	154	9.6–11	r/R	^124^
	*Steinchisma laxum*	2023	Colombia	280	11	R	^125^
	*Conyza canadensis*	2023	Mexico	42	13–20	R	^126^
	*Arctotheca calendula*	2025	Australia	78	10	R	^127^
Pro‐106‐Thr	*Eleusine indica*	2003	Malaysia	–	–	–	^128^
	*Lolium rigidum*	2012	Australia	208	6–11	r/R	^117^
	*Digitaria insularis*	2012	Brazil	64	2.3–3.9	r	^57^, ^129^
	*Echinochloa colona*	2016	Australia	88	1.2–2.2	r	^130^
	*Chloris virgata*	2020	Australia	210	4–27	r/R	^131^
Pro‐106‐Ala	*Lolium rigidum*	2007	Australia	96	9	r	^132^
	*Lolium perenne* ssp. *multiflorum*	2008	United States	37–168	2.4–15	r/R	^133^
	*Eleusine indica*	2021	China	281	1.8–8.2	r	^134^
Pro‐106‐Leu	*Echinochloa colona*	2016	Australia	88	1.1–2.2	r	^131^
	*Chloris virgata*	2016	Australia	515–594	2–9.7	r	^120^
Pro‐106‐His	*Digitaria sanguinalis*	2022	Argentina		5.1	r	^135^
Thr‐102‐Ser	*Tridax procumbens*	2018	Australia	142	3.9	r	^136^
Thr‐102‐Ile + Pro‐106‐Ser (TIPS)	*Eleusine indica*	2015	Malaysia	65	31	R	^137^
	*Bidens pilosa*	2016	Mexico	52	2.8–20	r/R	^138^
Thr‐102‐Ile + Pro‐106‐Thr (TIPT)	*Bidens subalternans*	2020	Paraguay	217	16–17	R	^139^
Thr‐102‐Ile + Ala‐103‐Val + Pro‐106‐Ser (TAP‐IVS)	*Amaranthus hybridus*	2019	Argentina	16.6	84	R	^140^, ^141^

Adapted from Gaines and Heap.^142^

^†^ Herbicide concentration causing a 50% reduction in biomass or mortality in the glyphosate‐susceptible population.

^‡^ Resistance factor of the resistant population(s) relative to the susceptible population.

^§^ Resistance level: r = moderate resistance (<10‐fold relative to the susceptible biotype) and R = high resistance (≥10‐fold).

Substitutions at position Pro‐106 are the most widely documented across both monocot and dicot GR weed species. At this position, Pro has been replaced by Ala, His, Leu, Thr, or, most commonly, Ser (Table 1).^142^ These mutations confer low levels of resistance (usually two to three times), but are sufficient for GR plants to survive the application of at least some recommended doses of glyphosate under field conditions.^33^ Structurally, Pro‐106 substitutions slightly narrow the glyphosate/PEP binding site cavity, thereby reducing glyphosate affinity without severely impairing enzyme function.^148^

Single mutations in Gly‐101 and Thr‐102 confer higher levels of resistance to glyphosate, but also reduce the volume of the PEP‐binding cavity, decreasing the enzyme's affinity for this natural substrate.^143^ These changes may negatively affect EPSPS efficiency and plant metabolism. Mutations at Thr‐102 were predicted to be unlikely to arise first or independently of Pro‐106 mutations, because a Thr‐102 alteration alone markedly reduces the enzyme's affinity for PEP.^33^ However, in *Tridax procumbens*, a single mutation was identified, replacing threonine with serine at position 102 of the *EPSPS* gene.^136^ Although this mutation reduces EPSPS efficiency, available evidence suggests that the associated fitness cost in the absence of glyphosate is not sufficient to compromise the persistence of GR biotypes.^149^

The accumulation of multiple mutations within the same EPSPS allele is well documented in glyphosate resistance.^150^ Although less frequent, multiple mutations have been found to confer high levels of glyphosate resistance. The double mutation known as TIPS (Thr‐102‐Ile + Pro‐106‐Ser) first evolved naturally in *E. indica* from Malaysia.^137^ Shortly afterwards, this mutation was also found in *Bidens pilosa* from Mexico.^138^ Double mutations generally take longer to reach high frequencies within a population;^33^ however, the occurrence of the TIPS mutation has become increasingly common. In addition, another double mutation variation, called TIPT (Thr‐102‐Ile + Pro‐106‐Thr), was found in *Bidens subalternans* from Paraguay.^139^ A TIPS *EPSPS* transgene is used for glyphosate resistance in GR maize, but, unlike the evolved TIPS EPSPS of weeds, the transgene construct possesses a promoter that provides a high level of gene expression.^11^

Another multiple mutation that confers high resistance to glyphosate is the TAP‐IVS triple mutation (Thr‐102‐Ile + Ala‐103‐Val + Pro‐106‐Ser). In addition to the already known negative effects of the mutations at positions 102 and 106 on the efficacy of the herbicide, the Val‐103‐Ala mutation increases the distance between the hydrogen bonds in the α‐helix of the EPSPS target site. This change causes the alanine side chain, which is longer than the valine side chain, to occupy more space and restrict the binding of glyphosate to the enzyme.^141^ This triple mutation was identified for the first time in a GR population of *Amaranthus hybridus* in Argentina.^140^ Although other cases of the TAP‐IVS mutation have been recorded in *A. hybridus* populations from Brazil and South Africa,^151^, ^152^ this triple mutation so far appears to be exclusive to this species.

Detailed analyses of both evolved and laboratory‐generated *EPSPS* mutations have clarified how changes in enzyme kinetics translate into glyphosate resistance and associated fitness effects.^11^, ^149^ In *A. hybridus*, comparisons of wild‐type and single, double, and triple field‐selected *EPSPS* variants, all assayed under identical conditions (Table 2),^153^ showed that increasing resistance is generally accompanied by reduced catalytic efficiency. Although reduced EPSPS efficiency would be expected to impose fitness constraints due to the high metabolic flux through the shikimate pathway, GR biotypes carrying the TAP‐IVS variant (three mutated codons and markedly reduced efficiency) showed no growth penalty when grown in isolation without glyphosate.^153^ However, under competition with wild‐type plants, these mutants produced fewer inflorescences, revealing a context‐dependent fitness cost that is expected to limit the persistence of highly impaired EPSPS variants in the absence of sustained glyphosate selection pressure.

**Table 2:** Kinetic parameters for *A. hyrbridus* EPSPS variants from the field

EPSPS	V_max_ (μmoles Pi min^−1^ mg^−1^)	Catalytic Efficiency			
		K_m_ (μM)	K_cat_ (min^−1^)	(min^−1^ mM^−1^)	glyphosate IC_50_ (μM)
Wild type	91a	115a	4.37	38	3.8a
P106S	62b	41b	2.98	73	97a
TIPS	9c	100a	0.43	4	3500b
TAP‐IVS	5.7c	26b	0.27	11	6500c

Adapted from Perotti *et al*.^153^. Different letters in columns indicated significant differences at the 95% level of confidence.

Monsanto scientists had argued that the odds of simultaneous, multiple mutations needed for a high level of EPSPS resistance occurring, while maintaining adequate catalytic activity, were astronomically improbable. However, they did not consider the possibility of creeping resistance, after which additional mutations accumulate in the surviving population and progressively enhance resistance, allowing survival, even at high application rates.^27^ This process has occurred through both NTSR and TSR mechanisms, which may co‐occur within the same biotype and, when combined, can confer resistance levels greater than the additive effects of each mechanism alone.

#### 3.2.2 EPSPS overproduction

The concentration of a molecular target in a plant, and the proportion that must be inhibited to cause lethality, are critical determinants of herbicide efficacy.^9^ EPSPS overproduction is an evolved mechanism of glyphosate resistance in which increased enzyme abundance allows sufficient EPSPS activity to be maintained despite partial inhibition by glyphosate, thereby sustaining flux through the shikimate pathway. This mechanism provides TSR without altering the structure or efficiency of the EPSPS enzyme.

EPSPS overproduction can result from gene amplification, typically characterized by an increased *EPSPS* gene copy number. Gene duplication can occur by different mechanisms, including tandem regions in the genome or extrachromosomal elements.^150^ Glyphosate resistance associated with EPSPS gene amplification was first reported in *A. palmeri* populations from the USA, representing the first documented case of any herbicide resistance caused by gene amplification.^154^ This mechanism has since been documented in several GR species (*Amaranthus tuberculatus*, *Bromus diandrus*, *Kochia scoparia*, *L. multiflorum*, and *Salsola tragus*). The level of resistance conferred by this mechanism is unpredictable, as it is directly correlated with the number of copies of the *EPSPS* gene, and can vary from three to five extra copies to more than 150.^155^ For example, in a study of 45 populations of *L. multiflorum*, the number of copies ranged from 11 to over 100.^156^ This mechanism does not seem to impose significant adaptive costs on GR plants.^157^ Additional EPSPS genes have been found at a single locus, multiple loci on the same chromosome, on multiple chromosomes, and as extra chromosomal genes.^158^

EPSPS overproduction can also result from the *EPSPS* overexpression without an increase in the gene copy number. For example, the most resistant lines of GR *L. rigidum* had both elevated levels of EPSPS mRNA and enzyme activity compared to the GS biotype 48 h after application of 1.5 kg ae ha^−1^ glyphosate, even though there was no gene duplication.^159^ This process can result from modifications to *cis* or *trans* regulatory elements, alterations to the gene's promoter region or post‐transcriptional mechanisms that increase the stability of the mRNA and reduce its degradation.^119^, ^160^ Sometimes, EPSPS activity can be elevated without increased *EPSPS* gene expression or mutated *EPSPS* gene. For example, a population of *E. colona* has higher basal EPSPS activity, but this increase was not due to the increased expression of the *EPSPS* gene.^160^ This mechanism has also been reported in *D. insularis* in Brazil,^161^ and *Leptochloa virgata* in Mexico.^119^

## 4 GENERAL ASPECTS OF GLYPHOSATE RESISTANCE IN WEEDS

Glyphosate resistance is a complex phenomenon that is highly dependent on biological, genetic, and environmental contexts, with its diversity and magnitude further influenced by factors such as growth stage, zygosity, and ploidy.^162^ For example, species of the genus *Conyza* differ in their sensitivity to glyphosate at the rosette stage, with *C. sumatrensis* being more sensitive than *C. bonariensis* and *C. canadensis*.^163^ In addition, for some GR weed biotypes, resistance may be found at some growth stages but not at others.^164^ Consequently, each species, population, and agronomic scenario may respond differently to the selection pressure imposed by glyphosate, contributing to an unprecedented diversity of resistance mechanisms for a single herbicide making generalizations difficult.

The relatively low resistance levels conferred by single *EPSPS* codon changes delayed the evolution of robust resistance.^34^ Consequently, glyphosate‐resistance alleles appear to be initially rare in native populations,^24^ highlighting the importance of seed hygiene and biosecurity to prevent their introduction and spread, rather than assuming that local evolution is the primary cause of the occurrence of GR weeds.^32^ A clear example occurred in Argentina, where the rapid spread of GR *S. halepense* was largely driven by the movement of contaminated agricultural machinery from north to south by a single grower. Although resistance was first detected in Salta province, GR *S. halepense* was soon reported throughout northern and central regions, highlighting long‐distance dispersal facilitated by human activity.^165^, ^166^ Similarly, GR *A. palmeri* with *EPSPS* amplification, detected in Europe, South America, and Asia, likely originated in the USA and were introduced *via* contaminated grain/seed lots (soybean and maize) or agricultural machinery.^167^, ^168^, ^169^, ^170^ These examples indicate that strengthening biosecurity measures to prevent the introduction of GR seeds or contaminated equipment may be more effective at limiting the spread of glyphosate resistance to new areas than relying solely on local resistance management, particularly during the early stages of resistance emergence.

Glyphosate dose also affects how resistance evolves.^32^ What constitutes a ‘high dose’ depends on plant growth stage, as a dose that is highly effective at the three‐leaf stage may be only marginally phytotoxic at later stages. For example, *C. bonariensis* was six‐fold more susceptible to glyphosate at the rosette stage than at flowering.^163^ Recommendations allowing glyphosate application at any growth stage often resulted in delayed treatments at effectively low doses, which can accelerate evolution of creeping resistance. In general, high herbicide doses tend to favor TSR, whereas low doses more often select NTSR, allowing survival and recombination of low‐level resistant individuals.^171^ This pattern may be less pronounced for glyphosate, because no single EPSPS codon change confers high‐level resistance. Dose effects are particularly relevant in cross‐pollinating or hybridizing genera such as *Amaranthus*, *Conyza*, and *Lolium*, where recombination facilitates the accumulation of multiple resistance mechanisms.^172^, ^173^, ^174^ In contrast, resistance evolution in self‐pollinating species is shaped primarily by mutation, selection, and limited gene flow, leading to independent resistance events associated with local management practices.^175^ This explains, for example, the high diversity of resistance mechanisms to glyphosate observed in *E. indica* across different regions worldwide.^176^

Selection for mutations providing low levels of glyphosate resistance can occur in fields sprayed with high glyphosate doses that would be lethal to some single‐mechanism GR biotypes, because many weeds are exposed to lower doses due to uneven herbicide coverage.^177^ By this means, creeping resistance can occur even at high application rates. Another proposed, but not yet fully established, effect of glyphosate is that some field doses may hormetically stimulate growth of GR weeds, enhancing their survival and propagation,^178^ as observed in *D. insularis* and *C. sumatrensis*.^179^, ^180^ Hormesis (the stimulatory effect of subtoxic doses of a toxin) is more commonly observed with glyphosate than with any other herbicide.^181^ However, this phenomenon needs further study, as most hormesis experiments do not characterize the underlying resistance mechanisms, nor are they conducted under field conditions.

Glyphosate resistance may also involve adaptive costs, including metabolic imbalances, reduced seed production, or slower growth, particularly in herbicide‐free environments.^149^ For example, TIPS and TAP‐IVS EPSPS mutations reduce EPSPS efficiency (Table 2),^153^ limiting aromatic amino acid synthesis and plant development.^182^ This deficiency of the TIPS EPSPS is offset in GR maize by the use of a powerful gene promoter.^11^ These costs may be ecological, physiological, and/or biochemical, depending on the species, genetic background, and environment. In *A. palmeri*, even high *EPSPS* gene copy numbers had no visible field penalties,^157^ whereas in *L. perenne*, significant seed production reductions were observed with gene amplification.^183^ In GR *A. tuberculatus*, extra EPSPS copies were associated with reductions in dry weight and lower intraspecific competitiveness.^184^ Thus, fitness costs vary with species, mechanism, and context. Discontinuing glyphosate use may reduce glyphosate resistance allele frequencies across generations, as cumulative costs manifest in the absence of selection.^181^

A major challenge arises from creeping resistance, driven by the accretion of resistance mechanisms under intense and prolonged selection pressure over vast areas for decades, particularly during the past three decades of GR crop cultivation. Because no single mutation provides robust resistance, populations progressively accumulate multiple TSR and/or NTSR traits over time, each conferring only weak resistance alone but higher resistance when combined.^162^, ^185^ Several well‐documented cases illustrate this phenomenon. For example, *D. insularis* combines TSR via the Thr‐106‐Ser mutation with NTSR mechanisms like reduced absorption, impaired translocation, and enhanced metabolism^57^; GR *E. colona* with AKR‐mediated metabolism together with a Pro‐106 *EPSPS* mutation^98^, ^100^; *E. indica* from Mexico and China, *A. palmeri* from Argentina and *Poa annua* from the USA, combining *EPSPS* amplification or overexpression with Pro‐106 mutations^69^, ^134^, ^186^, ^187^, ^188^; and *A. hybridus* in Brazil, where the triple TAP‐IVS mutation co‐occurs with EPSPS amplification.^152^, ^189^ In *C. canadensis*, separation of linked TSR and NTSR genes resulted in substantially lower resistance levels (seven‐ and nine‐fold, compared to the GS biotype, respectively) than when both mechanisms were present (45‐fold),^190^ demonstrating their synergistic effects. These examples show that the co‐occurrence of mechanisms can greatly enhance resistance and complicate weed management. Detecting one mechanism does not exclude others, underscoring the need for comprehensive characterization. The rise in reported cases of creeping glyphosate resistance reflects both more detailed studies of GR biotypes and the growing accumulation of TSR and NTSR traits under continuous selection pressure, increasingly evident in populations with multiple EPSPS mutations and/or combined mechanisms.

The accretion of resistance mechanisms can be either monogenic or polygenic. Monogenic resistance involves TSR, such as single EPSPS mutations^142^ or mutations combined with EPSPS amplification.^69^, ^134^, ^186^, ^187^, ^188^ This form of resistance typically evolves relatively rapidly but usually confers only low resistance levels. In contrast, the accumulation of multiple EPSPS mutations or increased EPSPS gene expression evolves more slowly but can result in higher resistance levels. Polygenic glyphosate resistance, which involves NTSR mechanisms alone or in combination with TSR (e.g., enhanced degradation together with mutated EPSPS), evolves gradually and can confer resistance ranging from low to high. Unlike polygenic resistance to other herbicides, commonly associated with broad NTSR mechanisms,^58^ polygenic glyphosate resistance does not confer multiple resistance to herbicides with other MoAs,^162^, ^191^ because glyphosate resistance traits are almost entirely glyphosate‐specific. Consequently, effective management of polygenic glyphosate resistance requires long‐term, integrated weed management strategies.^192^

## 5 ECONOMIC AND ENVIRONMENTAL IMPACTS OF GLYPHOSATE‐RESISTANT CROPS AND WEEDS

### 5.1 Economic and environmental benefits of glyphosate and GR crops

The economic impact of glyphosate, GR crops, and GR weeds has been profound. Before GR crops were introduced, glyphosate was an important herbicide, but had no strong effect on the economics of weed management. Extensive adoption of GR crops elevated glyphosate to the dominant herbicide worldwide, substantially reducing the market share and value of alternative herbicides,^39^ especially after it became generic in 2000. This dominance, in turn, slowed discovery and development of new herbicides with new MoAs.^9^, ^193^ Before the widespread evolution and spread of GR weeds, farmers growing GR crops experienced a ‘golden age’ of weed management, characterized by lower economic costs, high efficacy, and simplified weed control.^194^ Because glyphosate is a postemergence herbicide, GR cropping systems also enabled a marked reduction in tillage, thereby reducing soil erosion and use of fossil fuels.^39^, ^195^

The economic impacts of glyphosate extend beyond individual farms. The expansion of agricultural frontiers driven by transgenic crop adoption in developing countries facilitated the emergence of new agricultural regions, and even entire cities, that did not exist only a few decades ago. In Brazil, for example, no‐till systems were consolidated and expanded under GR crops,^196^ amplifying both economic and environmental benefits. As a result, glyphosate has supported, directly or indirectly, entire production chains, including pesticides, seeds, fertilizers, machinery, the food industry, consultancy, financing, research, logistics, and secondary and tertiary sectors within these emerging agricultural regions. These changes have improved rural quality of life and increased the availability of affordable food, which would likely be more costly in the absence of glyphosate and GR crops. In Brazil, agribusiness accounts for ~6.5% of gross domestic product from primary activities and about 23% when the entire supply chain is considered, raising the question of how much of this contribution derives from glyphosate‐dependent production systems.

### 5.2 Economic gains and losses associated with glyphosate resistance: the case of Brazil

**Figure 6:**
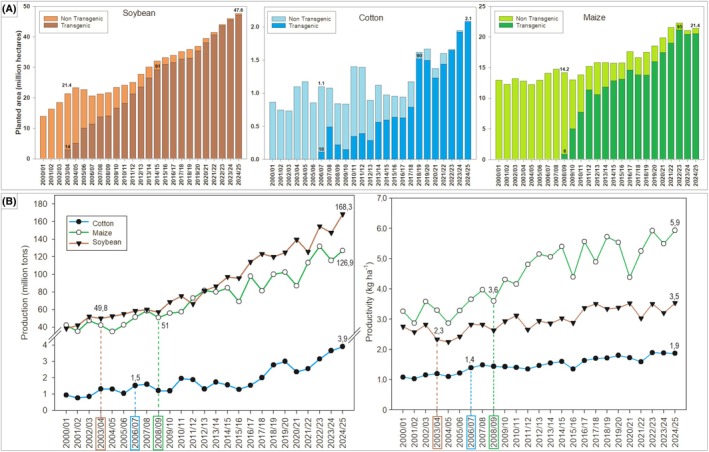
Trends in the adoption and production of transgenic crops in Brazil from the 2000/01 to the 2024/25 growing seasons. (A) Planted area and adoption percentage of trangenic cotton, maize, and soybean. (B) Total production and yield of cotton, maize, and soybean. The vertical line indicates the beginning of commercial cultivation of transgenic crops in Brazil. Adapted from Agbio Investor^12^ and CONAB.^198^

In Brazil, the cultivation and commercialization of transgenic crops were officially approved in 2005,^197^ although GR soybean had already been illegally cultivated since 2000; its legal cultivation began only in 2003 under an exceptional regulatory framework.^14^ Transgenic cotton and maize were subsequently introduced in the 2006/07 and 2008/09 seasons, respectively. Since then, the combined cultivated area of soybean, maize, and cotton has nearly doubled, increasing from 37 million ha in the early 2000s to 71 million ha in the 2024/25 season, coinciding with the progressive availability and adoption of transgenic cultivars. Adoption rates exceeded 90% for soybean, maize, and cotton by 2014, 2022, and 2019, respectively, and transgenic varieties currently dominate production systems for these crops (Fig. 6(A)–(C)).^12^, ^198^, ^200^ Over the same period, national production increased approximately 2.6‐fold for cotton, 2.5‐fold for maize, and 3.4‐fold for soybean (Fig. 6(D)), accompanied by yield gains of 74%, 61%, and 66%, respectively (Fig. 6(E)).^198^

The widespread adoption of glyphosate‐resistant (GR) crops has been a key component of Brazil's agricultural intensification and has contributed to its position as the world's largest producer and exporter of soybean, the second‐largest producer of maize, and the largest exporter of cotton. Despite the substantial economic and agronomic benefits, concerns have been raised regarding the potential role of GR crop adoption in accelerating the expansion of agriculture into natural areas. Although such land‐use change represents a potential environmental externality, attributing it directly to GR crops remains challenging, as broader economic, technological, and policy drivers also play major roles. In parallel, the rapid and widespread adoption of GR crops has substantially increased selection pressure on weed communities, reinforcing the need for coordinated resistance monitoring and long‐term assessment of weed management costs.

**Figure 7:**
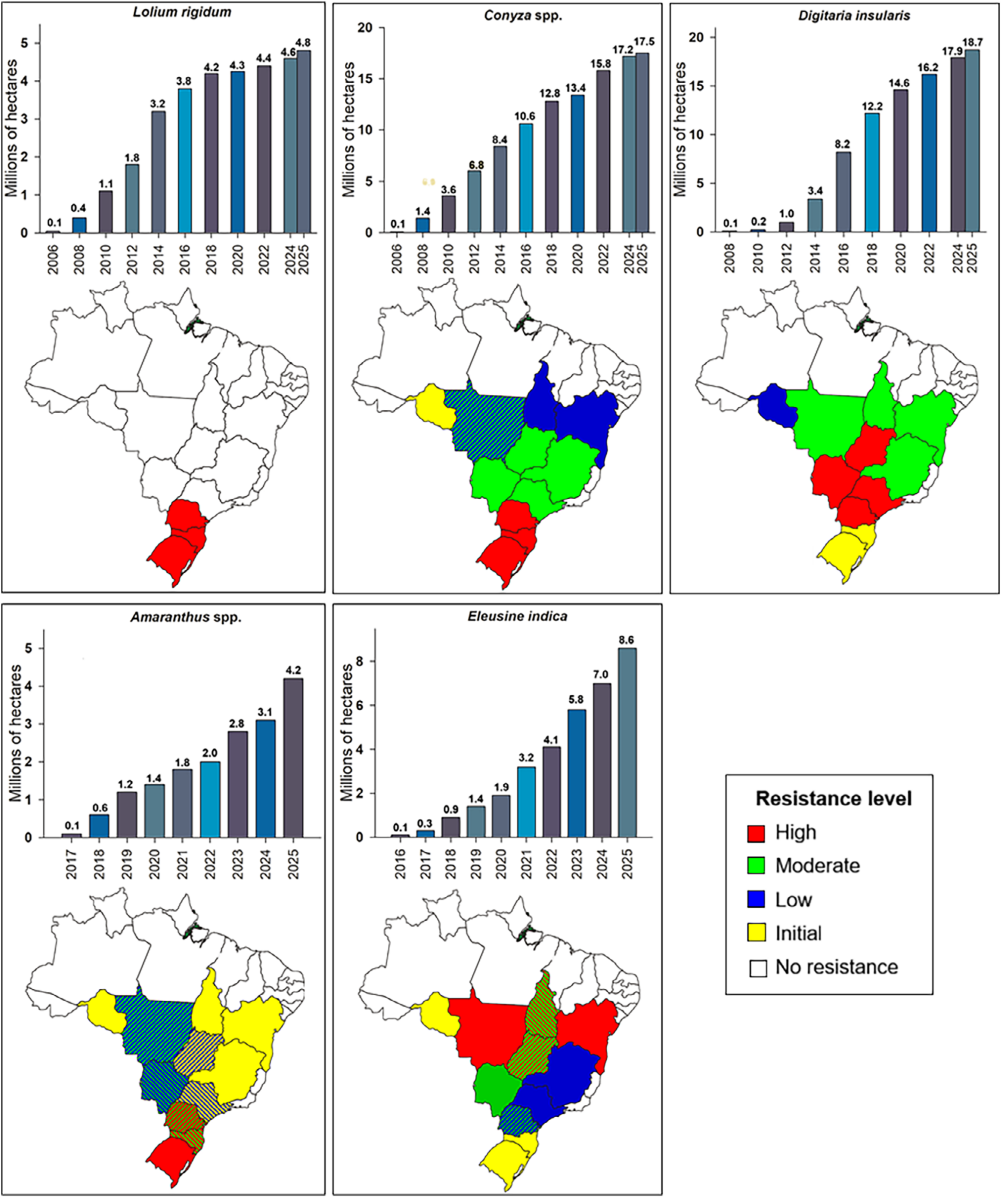
Temporal expansion and spatial distribution of glyphosate‐resistant weed species in Brazil. Bar charts show the estimated infested area (million hectares) over time for *Lolium rigidum, Conyza spp., Digitaria insularis, Amaranthus spp*., and *Eleusine indica*. Maps illustrate the regional distribution of resistance levels across Brazil during the 2024/25 cropping season. Adapted from GherbE^199^ and Adegas.^200^

The Herbology Research Group (GherbE) of Embrapa (Brazilian Agricultural Research Corporation) conducts continuous, coordinated national monitoring of herbicide resistance,^199^ providing quantitative evidence of the outcomes of prolonged glyphosate selection pressure in grain‐producing areas. Brazil currently reports 20 GR cases,^34^ predominantly involving *D. insulari*s, *L. multiflorum*, *E. indica*, and the species complexes of *Conyza spp*. and *Amaranthus spp*. GherbE data indicate a continuous increase in the area infested by these species (Fig. 7). *Lolium multiflorum* expanded gradually since 2006, reaching 4.8 million ha in 2025 and showing signs of stabilization. *Conyza spp*. expanded steadily to 17.5 million ha, whereas *D. insularis* exhibited the greatest expansion, from negligible levels in 2008 to 18.7 million ha in 2025. More recently, *Amaranthus spp*. increased from 0.1 to 4.2 million ha between 2017 and 2025, while *E. indica* expanded rapidly after 2019, reaching 8.6 million ha.

Beyond area expansion, glyphosate resistance has intensified spatially and in magnitude (Fig. 7). *Lolium multiflorum* is largely concentrated in southern Brazil with high phenotypic resistance levels; *Conyza spp*. are widely distributed, with high resistance in the South and moderate to low levels in the Central‐West and Southeast; and *D. insularis* represents the most critical scenario, combining extensive infested area with large regions of high resistance, particularly in the South and Southeast, while resistance levels in the Central‐West remain moderate. *Amaranthus spp*. show localized resistance hotspots, mainly in the South, whereas *E. indica* displays mosaics of moderate to high resistance in intensively cropped regions. Collectively, these patterns indicate a transition from early to advanced stages of resistance evolution, progressively eroding the effectiveness of glyphosate‐based weed management.

The economic consequences of glyphosate resistance are substantial and strongly species‐dependent. GherbE estimates from 2017 indicate average weed management cost increases of 57% for *L. multiflorum* (an annual species adapted to temperate climates), 82% for *Conyza spp*. (annual or biennial species with high dispersal capacity), and 235% for *D. insularis* (a perennial species reproducing both vegetatively and sexually), making it the costliest species overall.^48^ Where GR species co‐occur, costs increase dramatically, reaching 130% for *L. multiflorum* + *Conyza spp*. and up to 400% for *Conyza spp*. + *D. insularis*.^200^ With about 20 million ha infested in 2017, annual costs were estimated at US$ 1.5 billion (R$ 4.9 billion), potentially reaching US$ 2.8 billion (R$ 9.0 billion) when conservative 5% yield losses are included,^200^ based on the average exchange rate for 2017 (≈ R$ 3.2 per US dollar). These increases largely reflect the adoption of alternative herbicides, which are generally more expensive and sometimes less effective than glyphosate, as well as sharp increases (230–2650%) in sales of herbicides such as clethodim, haloxyfop‐methyl, triclopyr, glufosinate, 2,4‐D, diclosulam, and flumioxazin between 2010 and 2020.^31^

For the 2024/25 season, GherbE estimates indicate that at least 26 million ha (≈55% of the total Brazilian grain‐producing area) are affected by GR weed infestations.^199^ Although *D. insularis* has the greatest economic impact, *E. indica* (an annual species reproducing by seed and with high regrowth capacity) has emerged as one of the most challenging species to control,^176^ presenting the highest per‐hectare cost increase (approximately 420%). Total costs associated with *Conyza spp*. and *D. insularis* were estimated at US$ 0.77 billion (R$ 4.3 billion) and US$ 0.56 billion (R$ 3.1 billion), respectively, based on the 2025 average exchange rate (≈ R$ 5.6 per US dollar), whereas *E. indica* accounted for US$ 0.64 billion (R$ 3.6 billion), despite infesting roughly half the area of these species (Fig. 7). The total economic burden of glyphosate resistance in Brazil in 2025 was estimated at US$ 1.34 billion (R$ 7.5 billion), excluding yield losses; this value is lower than the simple sum of species‐specific estimates because infestations frequently occur in combination.

Apparent reductions in costs expressed in US dollars relative to 2017 largely reflect currency depreciation rather than a true reduction in the economic burden faced by farmers. Although increased competition and generic formulations have reduced unit prices for some alternative herbicides, overall weed management costs associated with glyphosate resistance remain high. Moreover, these estimates do not fully capture indirect costs related to mixed infestations, increased reliance on non‐chemical control practices (e.g., hand weeding and reduced tillage efficiency), management in other cropping systems (such as rice and perennial crops), the presence of volunteer plants, and intensified weed–crop competition. Overall, these data indicate that the economic impact of glyphosate resistance depends not only on the infested area, but also on species biology, resistance levels, and the practical limitations of available control options.

### 5.3 Erosion of environmental benefits and transition to new technologies

Numerous studies have reported potential environmental effects of glyphosate on specific non‐target organisms, frequently using exposure levels that exceed realistic field conditions.^201^ This review does not attempt to reassess this broad literature but emphasizes that the environmental impact of glyphosate must be evaluated relative to the herbicides and practices it replaced. During the first 25 years of GR crop adoption, herbicide use increased more rapidly in non‐GR systems than in GR systems,^202^ and GR crops contributed to reductions in both acute and chronic toxicity associated with herbicide use. The environmental impact quotients of herbicide programs in GR crops generally declined between 1996 and 2016.^37^ However, these benefits are being progressively eroded by the need to use additional herbicides to manage GR weeds.

As GR weeds have evolved and spread, the economic and environmental advantages of GR cropping systems are diminishing, requiring farmers to use additional herbicides and crops with stacked transgenes conferring resistance to other MoAs, such as glufosinate, dicamba, HPPD inhibitors, and 2,4‐D.^31^ Farmers have also increased reliance on pre‐plant herbicides and selective herbicides with older MoAs. The added costs of these measures, combined with yield losses where GR weeds are poorly controlled, have had major economic impacts in affected regions.^200^, ^203^ Even when available, implementing effective mixtures of herbicides with different MoAs to deal with the different resistances in a field can be prohibitively expensive.^204^

These challenges have driven increased investment in discovering and developing herbicides with new MoAs, as well as new herbicide‐resistant crops produced with transgenes and gene editing. In parallel, RNA interference (RNAi)–based approaches, long discussed as a potential tool for weed control but largely limited to proof‐of‐concept studies,^205^ have recently advanced toward practical application, including the first relevant demonstration of spray‐induced gene silencing targeting EPSPS in GR *D. insularis*.^206^ Numerous small companies have been started that offer new technologies to solve the herbicide resistance problem.^207^ The high cost of managing GR weeds has also accelerated the development of AI‐driven, robotic, precision herbicide application technologies, as well as non‐chemical precision systems (e.g., laser or robotic mechanical weeding). Glyphosate resistance has further reinforced the importance of integrated weed management strategies incorporating non‐chemical practices, including cover crops and crop rotations. All of these technologies are being developed in a context of climate change, which is expected to alter weed biology and competitive dynamics. Increased atmospheric CO_2_ can reduce the efficacy of glyphosate and other herbicides while increasing the vigor of C3 weeds more than that of C4 weeds and crops.^208^, ^209^ potentially shifting relative performance of different weed management strategies in future agroecosystems.

## 6 FINAL CONSIDERATIONS

With over 50 years of use and 30 years of both crop and weed resistance, glyphosate remains an essential tool for weed control and continues to be the most used herbicide globally. Compared with alternative weed management methods, glyphosate use, especially in GR crops, has provided environmental benefits, including reduced tillage, lower fossil fuel consumption, and a reduced environmental impact quotient.^36^, ^202^ However, the continued dominance and even the future use of this valuable asset are threatened by multiple factors, including expanding glyphosate resistance, perceived or real human toxicity, and new weed management technologies.

Glyphosate use remains controversial due to regulatory, public health, and environmental concerns, especially after the International Agency for Research on Cancer (IARC) of the World Health Organization classified glyphosate as ‘probably carcinogenic to humans’ in 2015 (Group 2A). This category also includes exposures such as art glass, indoor emissions from combusting wood or frying food, night shift work, consumption of red meat or hot beverages, talc, and cobalt metal.^210^ We chose these examples because most readers will be unfamiliar with other chemicals with this classification. The IARC decision has resulted in costly legal actions in the USA. However, regulatory agencies like the United States Environmental Protection Agency (USEPA) and the European Food Safety Authority (EFSA), along with regulatory authorities of many other countries, have not accepted the IARC conclusion and have continued to approve glyphosate after subsequent regulatory reviews. For example, in 2023, EFSA renewed approval of glyphosate for use until 2033. Extensive studies have found that both public and occupational exposures to glyphosate pose little risk to human health.^211^, ^212^ This conclusion is supported by large epidemiological studies that show no link between glyphosate exposure and cancer among pesticide applicators, except for a slight increase in acute myeloid leukemia risk in the highest exposure group, which the authors stated requires further confirmation.^213^ No verified molecular mechanism supports most proposed human toxicological claims,^214^ and hypotheses such as misincorporation of glyphosate in place of glycine in proteins have been experimentally refuted.^215^ Importantly, many toxicity studies reporting adverse effects rely on exposure levels far exceeding those encountered in food or the environment. Nonetheless, regulatory uncertainty and ongoing publication of toxicity studies continue to influence public perception and policy debates.

Experience from countries considering or implementing glyphosate bans highlights the potential consequences of premature regulatory decisions. In Sri Lanka, a ban motivated by health concerns led to severe declines in agricultural productivity and was subsequently partially reversed. In countries heavily dependent on GR crops, the impacts of a glyphosate ban would likely be even more severe in the absence of effective, economical alternatives. Mexico has proposed a glyphosate ban, but implementation has been delayed due to projected negative impacts on agricultural production.^22^ Until robust alternative weed management technologies are widely available, broad bans based on weak or inconsistent scientific evidence appear unlikely, although toxicological concerns will continue to challenge glyphosate use.

From an agronomic perspective, the evolution and spread of glyphosate resistance continue to accelerate due to the sustained and massive selection pressure imposed by its widespread use. This pressure not only promotes the emergence of resistance in new weed species but also drives creeping resistance through the gradual accumulation of both TSR and NTSR alleles in species already known to harbor GR biotypes. Although the global extent of glyphosate resistance remains poorly quantified, the continued reliance on glyphosate in both GR and non‐GR cropping systems indicates that many susceptible populations still persist. However, in the absence of widespread adoption of effective resistance management strategies, continued resistance evolution will inevitably erode the utility of glyphosate.

Sustaining glyphosate efficacy will require stronger resistance monitoring programs, standardized data collection, and continued research on resistance mechanisms, ecological interactions, and early detection tools. Integrated resistance management practices, including rotation and mixture of herbicides with different MoAs, use of crops with stacked resistance traits, crop rotation, intercropping, and non‐chemical control methods, are essential to slow resistance evolution and prolong glyphosate utility. While it is impossible to predict precisely when glyphosate use will decline substantially due to resistance, the increasing prevalence of GR weeds, together with the development of new herbicides and alternative weed management technologies, suggests that glyphosate use is likely to decrease within the next decade.

The agrochemical industry has made great efforts to discover new herbicides with properties comparable to glyphosate, but so far without success. At the same time, the development of non‐chemical weed management technologies has been driven both by societal aversion to chemical inputs and by the declining effectiveness of herbicides due to resistance. As chemical and non‐chemical technologies improve in efficacy and cost‐effectiveness, they are likely to contribute to a gradual reduction in reliance on glyphosate‐based weed management systems and GR crops. However, glyphosate has delivered exceptional weed management value across a wide range of agricultural and non‐agricultural systems for more than five decades, while GR crops have done so for three decades. Despite glyphosate being described as a ‘once‐in‐a‐century’ herbicide, whether glyphosate will remain in use for a full century is unclear.^38^, ^39^

## Data Availability

Data sharing not applicable to this article as no datasets were generated or analysed during the current study.
